# Scalariform-to-simple transition in vessel perforation plates triggered by differences in climate during the evolution of Adoxaceae

**DOI:** 10.1093/aob/mcw151

**Published:** 2016-08-07

**Authors:** Frederic Lens, Rutger A. Vos, Guillaume Charrier, Timo van der Niet, Vincent Merckx, Pieter Baas, Jesus Aguirre Gutierrez, Bart Jacobs, Larissa Chacon Dória, Erik Smets, Sylvain Delzon, Steven B. Janssens

**Affiliations:** ^1^Naturalis Biodiversity Center, Leiden University, P.O. Box 9517, 2300RA Leiden, The Netherlands; ^2^INRA, University of Bordeaux, UMR EGFV, F-33450 Talence, France; ^3^School of Life Sciences, University of Kwazulu-Natal, P. Bag X01, 3209, Scottsville, South Africa; ^4^Institute for Biodiversity and Ecosystem Dynamics, Computation Geo-Ecology, University of Amsterdam, Amsterdam, The Netherlands; ^5^Section Ecology, Evolution and Biodiversity Conservation, KU Leuven, Belgium; ^6^INRA, University of Bordeaux, UMR BIOGECO, F-33450 Talence, France; ^7^Botanic Garden Meise, Nieuwelaan 38, BE-1860 Meise, Belgium

**Keywords:** Adoxaceae, ancestral area and climate reconstruction, Baileyan wood trends, molecular dating, *Sambucus*, *Viburnum*, vessel perforation plate transition, wood anatomy

## Abstract

**Background and Aims** Angiosperms with simple vessel perforations have evolved many times independently of species having scalariform perforations, but detailed studies to understand why these transitions in wood evolution have happened are lacking. We focus on the striking difference in wood anatomy between two closely related genera of Adoxaceae, *Viburnum* and *Sambucus*, and link the anatomical divergence with climatic and physiological insights.

**Methods** After performing wood anatomical observations, we used a molecular phylogenetic framework to estimate divergence times for 127 Adoxaceae species. The conditions under which the genera diversified were estimated using ancestral area reconstruction and optimization of ancestral climates, and xylem-specific conductivity measurements were performed.

**Key Results** *Viburnum*, characterized by scalariform vessel perforations (ancestral), diversified earlier than *Sambucus*, having simple perforations (derived). Ancestral climate reconstruction analyses point to cold temperate preference for *Viburnum* and warm temperate for *Sambucus*. This is reflected in the xylem-specific conductivity rates of the co-occurring species investigated, showing that *Viburnum lantana* has rates much lower than *Sambucus nigra*.

**Conclusions** The lack of selective pressure for high conductive efficiency during early diversification of *Viburnum* and the potentially adaptive value of scalariform perforations in frost-prone cold temperate climates have led to retention of the ancestral vessel perforation type, while higher temperatures during early diversification of *Sambucus* have triggered the evolution of simple vessel perforations, allowing more efficient long-distance water transport.

## INTRODUCTION

Baileyan trends in wood anatomy are arguably one of the most common textbook examples of evolutionary patterns in plant anatomy. This assertion of a set of linear ancestral-to-derived transformation series in wood anatomical features found its origin in a broad comparison of the size of water-conductive cells in woody land plants (Bailey and Tupper, 1918). Later, Bailey’s student Frost (*1930a*, *b*) hypothesized that long and slender gymnosperm tracheids lost pit membranes in their scalariform end-wall pitting, and evolved into long, narrow angiosperm vessel elements with scalariform perforations including many bars (often >20). These vessel elements, considered ancestral within angiosperms due to their strong resemblance to tracheids of the gymnosperm outgroup, further developed into wider and thus hydraulically more efficient water conducting cells (Carlquist, 1975; Sperry *et al.*, 2007). In addition, vessel elements also shortened and at the same time the number of bars in the perforation plates reduced to zero, leading to short vessel elements with simple perforations. Interestingly, scalariform perforations were much more abundant in the Cretaceous than in the Tertiary (Wheeler and Baas, 1991), but in-depth palaeobotanical evidence fully supporting the Baileyan trends remains weak due to the scarcity of early woody angiosperms in the fossil record. For instance, two of the earliest vessel-bearing wood types are found in taxa with scalariform perforations (*Icacinoxylon*) or simple perforations (*Paraphyllantholoxylon*) (Wheeler and Lehman, 2009; Falcon-Lang *et al.*, 2012).

The scalariform-to-simple transition was established independently of any classification system, and was therefore hailed as a basis for identifying phylogenetic relationships among woody angiosperms (Bailey, 1957). However, Bailey and his students realized that vessel characters were prone to convergent evolution and that the use of homoplasious characters in the Tree of Life leads to erroneous conclusions (Bailey, 1944, 1957; confirmed in Baas and Wheeler, 1996). However, they never postulated a cause driving the transition, which is one of the major critiques of Bailey’s legacy (exhaustively discussed in Olson, 2012, 2014). It was only in the 1960s that Sherwin Carlquist placed the Baileyan trends in an adaptive framework. Carlquist pioneered the idea that evolution towards simple vessel perforations was driven by more hydraulic efficiency when plants moved from ever-wet or cold temperate habitats to (seasonally) dry habitats (Carlquist, 1966, 1975). Later studies confirmed Carlquist’s view, stating that scalariform perforations are retained in taxa that do not face selective pressure for high conductive efficiency, while a seasonal or permanent demand for great hydraulic efficiency in dry and/or warm areas has triggered species to evolve simple perforations, allowing more efficient long-distance water transport and thus more carbon fixation (e.g. Baas, 1976; Baas *et al.*, 1983; Jansen *et al.*, 2004). However, this scalariform-to-simple transition in vessel perforation plate morphology has been little tested in an evolutionary and ecophysiological framework (Sperry *et al.*, 2007; Christman and Sperry, 2010).

We assessed the dynamics of these scalariform-to-simple transitions within the large asterid clade (angiosperms), and selected the Adoxaceae genera *Viburnum* (∼165 species) and *Sambucus* (∼28 species) – two closely related taxa with strikingly different wood anatomy (Metcalfe and Chalk, 1950; Schweingruber, 1990) – as a case study in asterids. We first evaluated the direction of perforation plate transition using phylogenetic estimates from existing sequence data for a set of carefully sampled taxa among the asterids. Then, we integrated original wood anatomical observations of *Viburnum* and *Sambucus* with an updated molecular phylogeny based on existing and original sequence data from five markers (Eriksson and Donoghue, 1997; Clement *et al*., 2014). A divergence time estimation analysis on both genera and analyses unravelling ancestral areas and ancestral climate preferences were carried out on this combined molecular dataset to find out when and where *Viburnum* and *Sambucus* diverged from each other. Furthermore, we assessed whether present-day precipitation and temperature BIOCLIM variables (Hijmans *et al.*, 2005) can be linked to the wood anatomical variation observed, and we performed hydraulic conductivity measures in both genera to investigate our hypothesis that the scalariform-to-simple perforation plate shift is driven by peak conductive rates.

We set wished to test the following hypotheses: (1) simple-plated species have evolved many times independently within asterids from scalariform-plated relatives, and follow a unidirectional pattern; (2) *Viburnum* with scalariform perforations diversified first in habitats with low evaporative demands, while *Sambucus*, having simple vessel perforations, developed later, in hydraulically more demanding regions; (3) scalariform-to-simple perforation plate transitions are driven by selection acting on peak conductive rates; and (4) *Viburnum* and *Sambucus* differ dramatically in their wood anatomy, assuming a unique ecological niche for each of the genera based on present-day distribution patterns.

## MATERIALS AND METHODS

### Wood anatomy

**Fig. 1.**
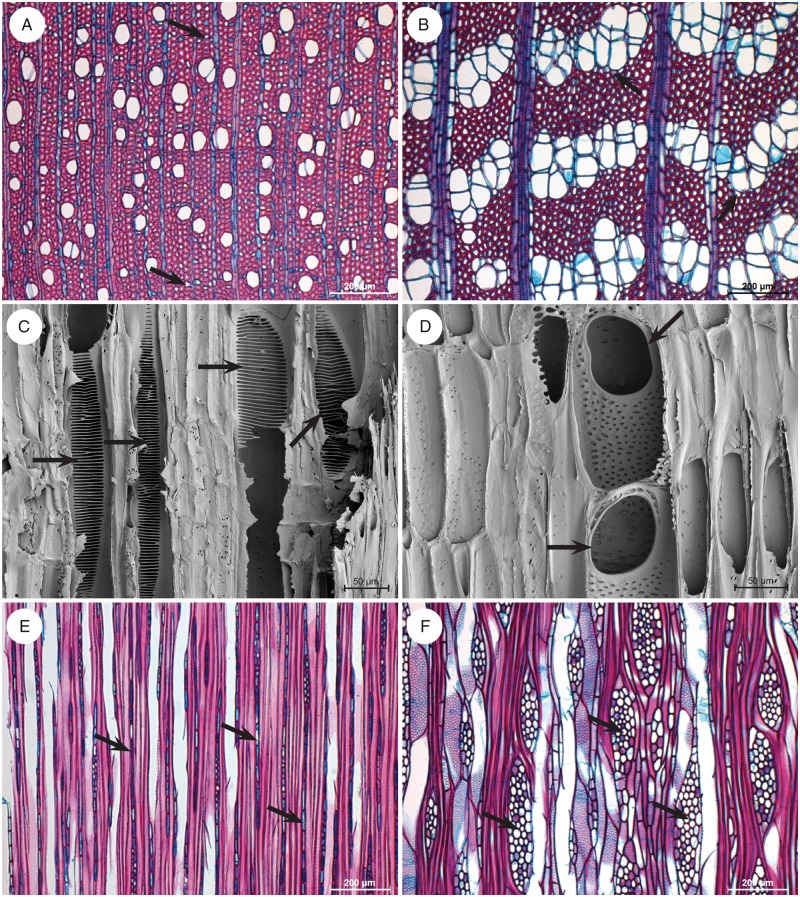
Illustrations of light microscope wood sections (A, B, E, F) and scanning electron microscope surfaces (C, D) showing the marked wood anatomical difference between *Viburnum* (A, C, E) and *Sambucus* (B, D, F). (A) *V. prunifolium*: transverse section, vessels mainly solitary, apotracheal axial parenchyma diffuse in aggregates (arrows). (B) *S. glauca*: transverse section, vessels in distinct clusters, axial parenchyma scanty paratracheal (arrows). (C) *V. furcatum*: radial section, scalariform vessel perforations with many bars (arrows). (D) *S. javanica*: radial section, simple vessel perforations (horizontal arrow). (E) *V. opulus*: tangential section, long multiseriate rays due to long uniseriate ends (arrows). (F) *S. glauca*: tangential section, multiseriate rays shorter and wider (arrows).

Wood descriptions of *Sambucus* and *Viburnum* are scattered in the literature, and most wood anatomical studies include only a limited number of species from a restricted geographical area (e.g. Moll and Janssonius, 1920; Kanehira, 1921; Metcalfe and Chalk, 1950; Ogata, 1988; Schweingruber, 1990; Benkova and Schweingruber, 2004; InsideWood, 2004 onwards). To expand existing data and to achieve a more representative sampling, we performed original wood anatomical observations of both genera, covering the entire distribution range and all major subclades according to the latest molecular phylogenies (Eriksson and Donoghue, 1997; Clement *et al*., 2014). In total, 44 wood specimens belonging to 17 *Sambuus* species and 17 *Viburnum* species were investigated using light microscopy and scanning electron microscopy (Fig. 1, Table 1, Supplementary Data Notes S1 and S2). The methodology of wood sectioning and slide preparation is described in Lens *et al.* (2005, 2007). In short, wood sections 25 μm thick were made using a sledge microtome (Reichert, Germany). After sectioning, the tissues were bleached with sodium hypochlorite and stained with a mixture of safranin and alcian blue (35:65), dehydrated with 50–75–96 % ethanol and mounted in euparal. Slides were observed using a Leica DM2500 light microscope and photographed with a Leica DFC-425C digital camera (Leica Microscopes, Germany). Detailed wood anatomical descriptions for *Viburnum* and *Sambucus* are available in Supplementary Data Note S2 and Table S1, and follow the IAWA list of microscopic features for hardwood identification (IAWA Committee, 1989). For the terminology of the imperforate elements, we tend to agree with Carlquist (1984), who links the vessel distribution pattern with the presumed water-conducting capacity of the imperforate elements. Therefore, we prefer to name the imperforate elements in the ground tissue of *Viburnum* ‘tracheids’ rather than ‘fibres with distinctly bordered pits’, although more experimental studies in *Viburnum* should be carried out to support this statement.

**Table 1.** Overview of wood anatomical characters summarizing the most important differences between *Sambucus* and *Viburnum*

Distinguishing wood features	*Viburnum*	*Sambucus*
Vessel perforation type	scalariform (often >20 bars)	Simple
Vessel element length (µm)	700–1600	300–500
Vessel distribution	Vessels solitary: VGI <1·5	Vessels grouped: 2 < VGI < 5
Intervessel pits	Opposite, scalariform or a mixture of both	Alternate
Vascular tracheids	–	+
Imperforate tracheary elements in the ground tissue	Elements with distinctly bordered pits	Elements with simple to minutely bordered pits
Axial parenchyma distribution	Diffuse apotracheal or diffuse in aggregates	Scanty paratracheal
Number of cells per axial parenchyma strand	Often 5–8	Often 3 or 4
Vessel-ray pitting	Distinct borders	Reduced borders
Number of rows of marginal ray cells per multiseriate ray	Variable: 1–15	1 or 2
Multiseriate ray height (µm)	Often 600–2500	400–600
Uniseriate ray density (mm^−1^)	Often 3–10	0–2

VGI, Vessel Grouping Index.

**Fig. 2.**
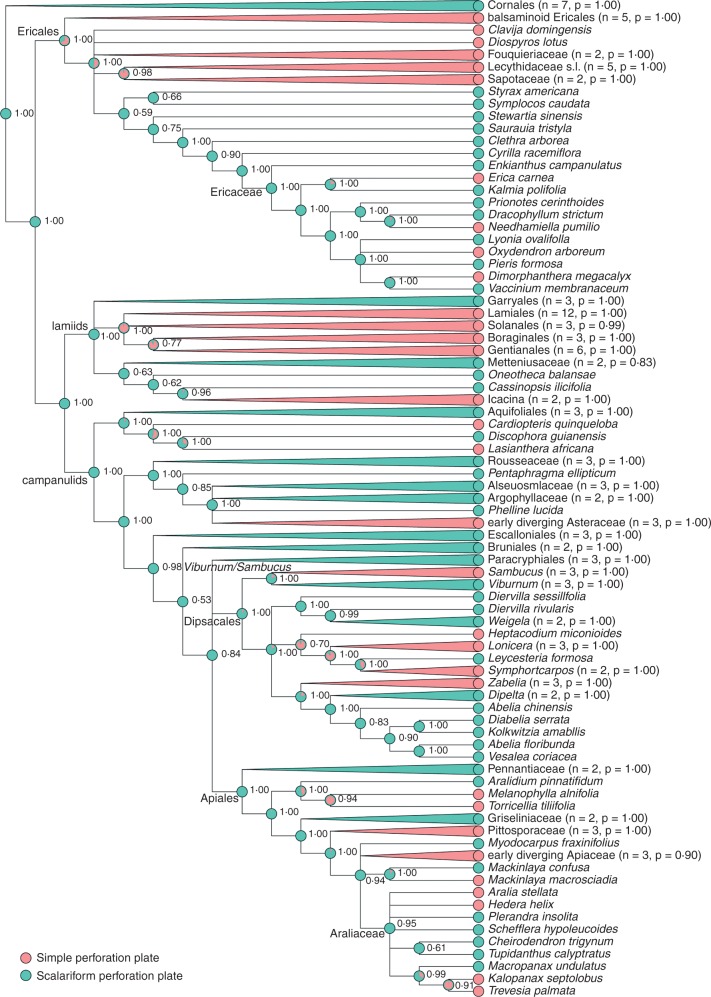
Majority-rule consensus tree topology summarizing the Bayesian comparative analysis of the evolutionary dynamics of transitions between scalariform (green) and simple (red) perforation plate types in asterids. Values on interior nodes are clade posterior probabilities. Pie charts on interior nodes are ancestral state reconstructions proportional to the probability of the respective reconstruction. Clades that are monophyletic as well as uniform in their character state are shown collapsed; the number of subtended species and the posterior probability of the root of the clade are given in parentheses. The scalariform plate perforation type is reconstructed as ancestral, with numerous independent transitions to the simple plate perforation type (at least 10), but note the apparent reversal for *Leycesteria.*

**Fig. 3.**
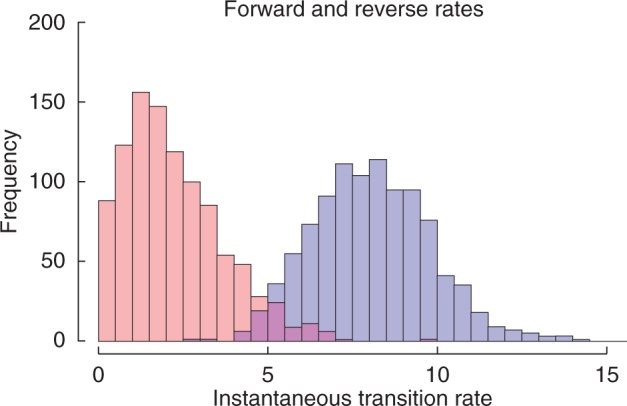
Distributions of instantaneous transition rates between scalariform and simple vessel perforation plates. Estimates of instantaneous transition rates from scalariform to simple (forward rates, violet) and simple to scalariform (reverse rates, salmon) across 10^6^ iterations (less 10 k burn-in) of MCMC ancestral state reconstruction using BayesTraits on the posterior tree sample (*n* = 722) for the asterids. Transitions from scalariform to simple occur at significantly higher rates than reversals, and the latter are not significantly different from zero.

To assess the polarity of transitions between vessel perforation plates, we applied a Bayesian framework, using estimates of the phylogeny of 152 carefully selected asterid species, taking into account the variation in vessel perforation plate morphology across the asterids (Figs 2 and 3). We want to stress that we only intend to provide a realistic (but not final) estimation of the number of shifts in perforation plates in this huge clade, including about 100 000 (woody and herbaceous) species. Therefore, we used the SUPERSMART pipeline v.0.1.22 (Antonelli *et al.*, 2014) to identify commonly sequenced markers and representative species with sufficient published sequence coverage (*matK*, *n* *=* 133; *ndhF*, *n* *=* 121; *rbcL*, *n* *=* 110 and *rps16*, *n* *=* 71; accession numbers are given in Supplementary Data Table S2). We aligned the markers using MAFFT v. 7.130b (Katoh *et al.*, 2002) with default settings and autoselected alignment strategy, which chose FFT-NS-i for *matK*, *ndhF* and *rbcL*, and L-INS-i for *rps16*. We concatenated the alignments into a supermatrix and analysed this using ExaBayes v. 1.4.1 under default settings (Aberer *et al.*, 2014) to construct a posterior sample of trees, which we rooted on Cornales.

We used our posterior sample of trees to run comparative analyses of transitions between scalariform and simple using BayesTraits v. 2.0 (Meade and Pagel, 2014), which we ran as Markov chain Monte Carlo (MCMC) analyses using an exponential prior for the transition rates and using a stepping stone sampler (Xie *et al.*, 2011) to obtain estimates of the marginal likelihoods. We did this for different instantaneous transition rate matrices: unconstrained; transition rate from simple to scalariform set to 0, i.e. no reversals; and forward and reverse rates set to equal. We also ran an MCMC analysis to reconstruct ancestral state distributions for all interior nodes

### Taxon sampling phylogenetic analysis

We expanded existing phylogenetic studies for *Sambucus* based on original sequence data of five molecular markers that had already been used in several published *Viburnum* phylogenies (*trnK*, *matK*, *trnS*-*G*, *atpB*-*rbcL*, internal transcribed spacer (ITS). The newly generated *Sambucus* sequences were combined with the published sequences of *Viburnum* and the remaining genera of the Adoxaceae (*Adoxa*, *Sinadoxa* and *Tetradoxa*; Eriksson and Donoghue, 1997; Winkworth and Donoghue, 2005; Clement and Donoghue, 2011, 2012; Chatelet *et al.*, 2013; Schmerler *et al.*, 2012; Clement *et al*., 2014). In our sampling, *Sambucus* is represented by 27 species, *Viburnum* by 97 species and the small herbaceous genera *Adoxa*, *Sinadoxa* and *Tetradoxa* by one species each (see Note S1 for detailed species list). Members of the sister family Caprifoliaceae s.l., *Weigela praecox* and *Diervilla sessilifolia*, were selected as outgroup.

### Species, subspecies and synonyms in *Sambucus*

The number of species in *Sambucus* is still under debate. During his revisions of the genus, von Schwerin (1909, 1920) reduced the number of *Sambucus* species from over 100 to 28. A more recent revision by Bolli (1994) further reduced the number of taxonomically valid species names to nine. However, Bolli’s morphological species concept remains ambiguous and needs to be adjusted, which was later confirmed by molecular phylogenetic studies (Eriksson and Donoghue, 1997; Clarke and Tobutt, 2006). An aim of the present study is to further contribute to clarifying species relationships within *Sambucus*, but fully resolving species boundaries is beyond the scope of this paper.

### Molecular protocols and sequence analyses

DNA isolation followed the protocol of Janssens *et al.* (2006, 2009), whereas amplification of *trnK*, *matK*, *trnS*-*G*, *atpB*-*rbcL* and ITS was carried out following Young *et al.* (1999), Clement and Donoghue (2011), Manen *et al.* (1994) and White *et al.* (1990), respectively. Contiguous sequences were assembled using Geneious v. 7.0.6 (Biomatters, New Zealand). Automatic alignments were carried with MAFFT (Katoh *et al.*, 2002) under an E-INS-i algorithm. Subsequent manual fine-tuning of the aligned dataset was done in Geneious v. 7.0.6. Gaps were treated as missing data, whereas potentially informative insertions and deletions were coded according to the ‘simple indel coding’ method of Simmons and Ochoterena (2000). Analyses performed without gap coding did not change the topology.

The best-fit nucleotide substitution model for each plastid and nuclear dataset was determined using jModelTest 2.1.4. (Posada, 2008) under the Akaike information criterion (AIC). The GTR + I + G model was found as best fit for *trnS-G* and *trnK*, whereas the GTR + G model was calculated as best substitution model for *matK* and ITS, and F81 + I as best substitution model for *atpB-rbcL*. A mixed-model approach was used in which the combined dataset was partitioned in order to apply a different model of evolution on each DNA region (Ronquist and Huelsenbeck, 2003). Bayesian inference analyses were conducted with MrBayes v. 3.1 (Huelsenbeck and Ronquist, 2001) on five individual data partitions and a combined data matrix. Each analysis was run twice for 10 million generations. Trees were sampled every 2500 generations. Inspection of chain convergence and effective sample size (ESS) parameters was done with TRACER v. 1.4 (Rambaut and Drummond, 2007). Only Bayesian posterior probabilities (BPPs) above 0·95 were taken into consideration (Suzuki *et al.*, 2002).

### Divergence time analysis

**Fig. 4.**
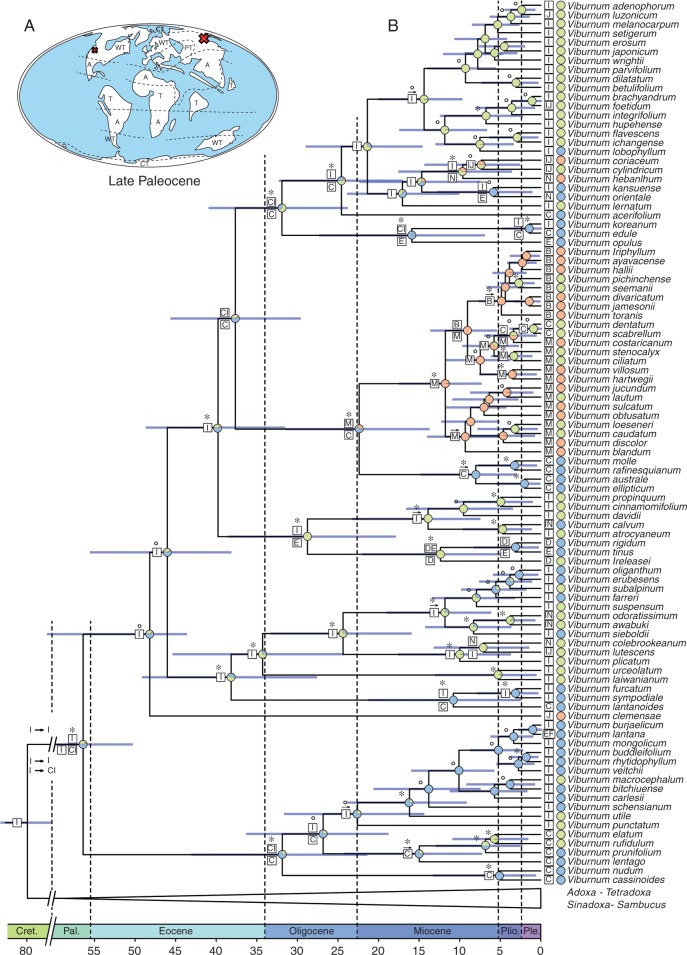

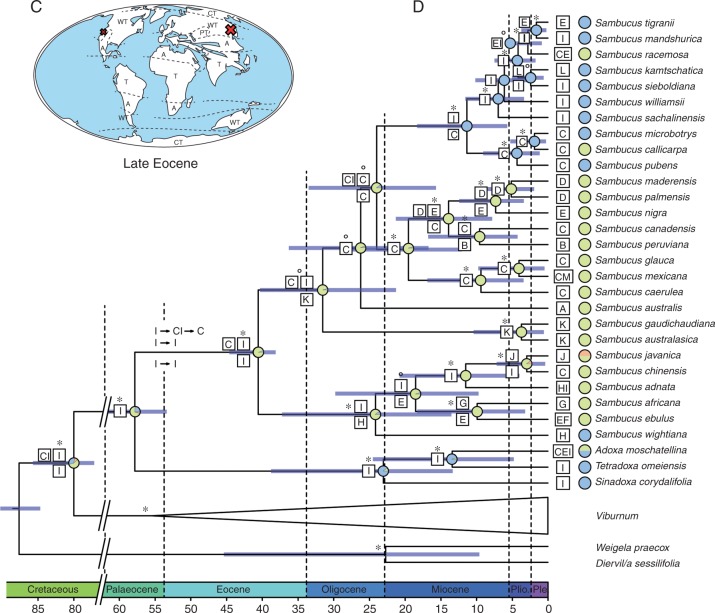
Palaeomaps and maximum clade credibility phylogeny for *Viburnum* (A, B) and *Sambucus* (C, D) inferred from combined ITS, *trnK*, *matK*, *atpB*-*rbcL* and *trnS-G* as obtained from BEAST. (A, C) Palaeomap of *Viburnum* (A) and *Sambucus* (C) during initial diversification of both genera. The red X marks the putative area(s) of initial diversification. A, Arid; WT, warm temperate; CT, cold temperate; T, tropical; PT, paratropical. (B, D) Maximum clade credibility phylogeny showing results from ancestral area reconstruction and ancestral climate reconstruction. Bayesian posterior probabilities (BPPs) are displayed above branches; asterisks indicate a BPP value ≥0·95, whereas BPP values between 0·5 and 0·95 are indicated by circles. Ancestral area reconstructions (AARs) with the highest likelihood value are shown as boxes at each node. A single box refers to a specific distribution range, whereas multiple boxes occurring either above or below indicate alternative AARs. The following abbreviations are used: A, South American Central Atlantic; B, South American Andes; C, North America; D, Macaronesia; E, Europe; F, North African Mediterranean and Atlas; G, Montane Eastern Africa; H, Western Himalaya; I, Subtropical-Temperate East Asia; J, Malesia and Indochina; K, Western Australia; L, Northeastern Siberia and Kamchatka; M, Caribbean; N, Western India and Sri Lanka. Pie charts at each node show the posterior probability of each possible climate type for the ancestral climate preference reconstruction (blue, cold temperate climate; green, warm temperate climate; orange, tropical climate).

The node ages within the Adoxaceae were estimated based on a calibrated ultrametric phylogenetic tree (Fig. 4). The tree was calibrated using a combination of primary calibration points based on the ages of well-identified fossils available for *Sambucus* and *Viburnum*, and a secondary calibration point for the age of Adoxaceae from a previous dating analysis (Janssens *et al.*, 2009). Three calibration points were used for age estimation: (1) minimum crown age constrained at 38 million years ago (Ma) for *Sambucus* (crown age) based on fossil endocarps from the late Eocene to Pliocene found in Europe (Reid and Chandler, 1926); (2) crown age of *Viburnum* constrained at a minimum age of 47·8 Ma, corresponding to the report of fossil leaves from the middle Eocene Jijuntun formation (Wang *et al.*, 2010); and (3) Adoxaceae crown node set at 79·9 Ma, based on the large asterid analysis of Janssens *et al.* (2009), which matched the dating analysis of Bremer *et al.* (2004). The two fossil calibration points used in this study were modelled in BEAST v. 1.8.0 under a log-normal distribution (Drummond and Rambaut, 2007), with an offset that equals the age of the fossil calibration point, a mean of 1·0 and a standard deviation of 1·0. The third calibration point was given a normal distribution with a mean value and standard deviation of 5·0 (cf. Janssens *et al.*, 2009).

A χ^2^ likelihood ratio test, used to assess rate heterogeneity among lineages (Felsenstein, 1988), indicated that the substitution rates in the combined dataset are not clock-like (*P* <0·001 for all markers). We therefore used a Bayesian approach as implemented in BEAST to calculate divergence times. To initiate the Bayesian dating analysis and to cope with the zero likelihood issue in BEAST, we used a starting tree that was obtained by carrying out a maximum likelihood analysis on the combined dataset in RAxML 7.2.8 (Stamatakis *et al.*, 2008) under a GTR + G model with the rooted likelihood tree as input tree for a penalized likelihood analysis in r8s 1·70 (Sanderson, 2004), with all calibration points used as described above. Because of differences in substitution models among the individual chloroplast genes and ITS, we performed a partitioned Bayesian MCMC analysis under the assumption of the Yule speciation model and a relaxed log-normal clock. Partitions were unlinked for the model of DNA sequence evolution. All other priors were kept as defaults. Two runs of 20 million generations were performed, sampling every 2000 generations. Convergence of the chains (the ESS parameter exceeding 200) was carried out with TRACER v.1.6 (Rambaut and Drummond, 2007). The two runs were combined, discarding the initial 2 million generations as burn-in using Logcombiner v. 1.8.0, and a maximum clade credibility tree using a posterior probability limit of 0·5 was calculated using TreeAnnotator v.1.8.0 (Drummond and Rambaut, 2007).

### Ancestral area reconstruction

Reconstruction of ancestral distribution ranges was carried out following the dispersal–extinction–cladogenesis model of Lagrange (Ree *et al.*, 2005; Ree and Smith, 2008; Fig. 4). Lagrange scripts were generated using the online Lagrange configurator (www.reelab.net/lagrange/configurator/index). The maximum clade credibility tree obtained from the dating analysis with BEAST v. 1.8.0 was used as input tree. In total, 14 areas were defined based on known distribution ranges of species within the Adoxaceae and delimited using Takhtajan’s (1986) floristic regions of the world (Fig. 4). Maximum range size was defined at 2 (only three out of 141 taxa investigated in the ancestral area analysis occur in more than one distribution area, and were also set at 2). Dispersal effectiveness was set at ‘symmetric’, but configured between each distribution area. Whereas adjacent regions were set at 1·0, dispersal effectiveness was set at 0·7 between non-adjacent regions on the same continent and adjacent regions on different continents. Dispersal effectiveness was set at 0·2 between non-adjacent regions on different continents.

Due to the uncertain phylogenetic position of *V. clemensae* among the earlier-diverging lineages of *Viburnum*, we ran an alternative ancestral area reconstruction analysis in which *V. clemensae* was constrained as sister species to all other *Viburnum* species (cf. Moore and Donoghue, 2007; Schmerler *et al.*, 2012). All parameters used were the same as in our original analysis.

### Ancestral climate preference reconstruction

Bayesian stochastic character mapping (BSCM) as implemented in SIMMAP 1.5 was applied to reconstruct the ancestral preference in climate conditions in Adoxaceae (Bollback, 2006; Fig. 4). Climate preference was scored as follows: (1) tropical; (2) warm temperate; and (3) cold temperate. Distribution data from the Global Biodiversity Information Facility (GBIF) were used to assess climate types, after being screened for erroneous localities. The GBIF coordinates obtained from species investigated were plotted on the updated Köppen–Geiger climate classification (Kottek *et al.*, 2006) using DIVA-GIS (Hijmans *et al.*, 2011). Next, species were scored in one of the broad climate types according to the climate zone that corresponds to the plotted distribution range of each species analysed. In addition, elevation ranges were taken into consideration to assess the exact climate preference for each species. In order to guarantee a uniform method for evaluating the climate preference for all our Adoxaceae accessions, thereby avoiding putative bias that could affect the results of our overall climate preference analysis, we ignored the Schmerler *et al.* (2012) GBIF dataset for *Viburnum* and built our own.

Climate types are regarded as unordered with weight 1. Five thousand randomly sampled Bayesian topologies from the BEAST output file [generated by the ‘sub-sample tree’ function in BayesRate 1·5 (Silvestro *et al.*, 2011)] were used as topology input. Hyperparameters defining the mean (*E*) and standard deviation accommodates the bias rate parameter *I* and the substitution rate parameter *θ*. A flat prior was used for the bias rate parameter *I* in all analyses. Due to the significant influence of *E* (*θ*) and standard deviation. (*θ*) on the overall outcome of each analysis, they were independently selected for each character using the ‘number of realizations sampled from priors’ function as implemented in SIMMAP (Couvreur *et al.*, 2008; Janssens *et al.*, 2012). Once the optimized hyperparameters are estimated, the impact of the standard deviation (*θ*) value becomes insignificant and the mean *E* (*θ*) value can be optimized for each character. As a result, the standard deviation (*θ*) is set at 2. We also carried out an alternative climate preference reconstruction in which *V. clemensae* was constrained as sister species to all remaining *Viburnum* species using the same parameters.

### Present climate preference analysis

A phylogenetically broad selection of geographic locations obtained from the GBIF website for *Viburnum* and *Sambucus* species was analysed using principal components analysis (PCA). This PCA was used as a visualization tool to assess potential differences between the ecological niches occupied by the two genera based on present-day distribution data (using the 19 scaled/centred WorldClim bioclimatic variables; Hijmans *et al.*, 2005). In total, 12 483 geographic locations from 30 *Viburnum* species were analysed. For *Sambucus*, 16 746 locations from 14 species were included in the PCA (see Supplementary Data Fig. S1 for species list). For *Sambucus* *nigra*, a random set of 1000 geographic locations from its full distribution was taken from the 206 000 that were available, preventing the niche of the genus from being represented by largely one species.

### Xylem specific conductivity (*K*_s_) measurements

Six equally sized (>1 m in length, 7–9 mm in diameter), leaf-bearing branches of *Viburnum lantana* and *Sambucus nigra* were sampled in the neighbourhood of Bordeaux in a public park in Gradignan (branches about 4–5 years old) and a more mesic forest in Madirac (branches about 2–3 years old; France), respectively, immediately put in a sealed plastic bag after removing the leaves, and stored in a refrigerator overnight. First, air was injected at one side of the branch at 2 bars to assess the maximum vessel length for both species. Then, we trimmed stem segments corresponding to ∼70 % open vessels under water (40 cm for *V. lantana* and 10 cm for *S. nigra*), and flushed the stems with 2 bars for 15–30 min using a solution of 10 mm KCl and 1 mm CaCl_2_ with the XYL’em apparatus (Bronkhorst, Montigny-les-Cormeilles, France). The maximum specific conductivity was determined based on the gravity method (Alder *et al.*, 1997) using the software program graviflow v. 1.1 with four increasing pressure heads (1–3 kPa for each 10-cm branch, 3–5 kPa for each 40-cm branch). Conductance was calculated as the slope of the relation between flow rate and pressure gradient. After the measurements, cross-sections were made and photographed (according to the method described above in the section Wood anatomy) to calculate specific conductivity (*K*_s_), by multiplying by sample length and dividing by xylem area. Feret diameter of each individual vessel and vessel density was measured by creating a binary image with an adjusted threshold and using the ‘analyze particles’ function of ImageJ software (http://rsb.info.nih.gov/ij) for about 3000 vessels per section (five sections per species).

## RESULTS

### Wood anatomy of *Sambucus* and *Viburnum*, and broad phylogenetic comparative analysis of vessel perforation plates in asterids

The wood anatomical descriptions of *Sambucus* and *Viburnum* are summarized in Notes S1 and S2 and Table 1, and illustrated in Fig. 1. Character coding of the perforation states for the broadly sampled asterids and the accession numbers of sequences used in their phylogenetic analysis are shown in Table S2.

The Bayesian phylogenetic supermatrix analysis for the broadly sampled asterids converged quickly and resulted in a posterior sample (*n* *=* 722, after discarding burn-in of 10 %) that implied well-supported clades [mean posterior probability (PP) on nodes under all-compatible consensus, i.e. on a fully resolved tree, 0·85 ± 0·26] that conform well to the common understanding of the systematics of this group. The ancestral state reconstructions suggest that the scalariform perforation type is ancestral for the asterids as a whole, as well as within the Paracryphiales–Dipsacales clade, and that there are three shifts in the latter towards simple perforations: one leading to *Sambucus*; one leading to *Heptacodium* and relatives (Caprifoliaceae) and one to *Zabelia* (Fig. 2).

Within Caprifoliaceae there is evidence for one reversal back to scalariform perforations in *Leycesteria*. However, constraining simple-to-scalariform reversals to a rate of 0 (i.e. no reversals, marginal lnL −76·079555) is overall statistically indistinguishable from the unconstrained model [marginal lnL −76·811068; in a Bayes factor (BF) analysis the test statistic is the absolute difference between these marginal likelihoods, which in this case does not exceed 1, i.e. not significant]. In contrast, scalariform-to-simple transitions far exceed reversals (Fig. 3), such that constraining forward and reverse transitions rates to equal yields a significantly poorer fit (marginal lnL -79·150205, BF=2·339137 compared with the unconstrained model).

### Molecular phylogenetics of Adoxaceae

Since the phylogenetic relationships within *Viburnum* and *Sambucus* are not the focus of this paper, we list here the main results briefly, and comment in more detail about the relationships in Supplementary Data Note S3. *Viburnum* is sister to all remaining Adoxaceae genera and *Sambucus* is sister to the clade containing *Adoxa*, *Sinadoxa* and *Tetradoxa* (cf. previous papers listed in Note S3; Fig. 4). Our *Viburnum* topology supports previous work (cf. previous papers listed in Note S3), except for the position of *Viburnum* section *Valvatotinus sensu* Winkworth and Donoghue (2005), which comes out as the earliest-diverging clade in our study. Studies by Donoghue and collaborators fix *V. clemensae* as root prior, making this species sister to the remaining *Viburnum* species. In *Sambucus*, species of the section *Ebulus sensu* Hara (1983) are sister to the remainder of the genus (Fig. 4). The rest of the clade contains the monophyletic *Sambucus* section *Botryosambucus* and the paraphyletic section *Sambucus sensu* Bolli (1994).

### Divergence time estimates

After 20 million generations, all parameters and age estimations had reached ESS values of >200. Adoxaceae were estimated to have a range in crown node age of 75·1–86·4 Ma [95 % highest posterior density (HPD), mean age 80.6 Ma, calibration point used 79·9 Ma]. This suggests an origin in the Late Cretaceous (Fig. 4). The range in crown node age for *Viburnum* was estimated to be 49·9 – 63·0 Ma (95 % HPD, mean age 56·1 Ma, calibration point used 47·8 Ma; Late Palaeocene), whereas its stem node age is similar to the crown node age of the Adoxaceae. The split of *Sambucus* and the lineage towards *Adoxa*, *Tetradoxa* and *Sinadoxa* occurred in the Palaeocene, with an estimated mean age of 58·4 Ma (95 % HPD 48·8 – 66·1 Ma). Diversification of *Sambucus* occurred in the Late Eocene at an estimated mean age of 40·9 Ma (95 % HPD 38·4 – 44·9 Ma). At the end of the Oligocene, *Sinadoxa* split off from the ancestor of *Adoxa* and *Tetradoxa* (95 % HPD 13·4–39·1 Ma, mean age estimate 23·2 Ma), whereas *Adoxa* diversified from *Tetradoxa* in the Miocene (95 % HPD 4·8–24·7 Ma, mean age estimate 13·5 Ma).

Within *Sambucus*, most of the diversification occurred between 20 and 4 Ma (Fig. 4). The initial diversification in the genus in the Late Eocene resulted in two major lineages (species of section *Ebulus*, and species of sections *Sambucus* and *Botryosambucus*, respectively), which in turn started to diversify during the Oligocene (*Sambucus*, 95 % HPD 13·6–37·5 Ma and mean age estimate 24·3 Ma; *Botryosambucus*, 95 % HPD 21·4 – 40·6 Ma and mean age estimate 31·7 Ma). The *Sambucus* clade that contains species from sections *Sambucus* and *Botryosambucus* is characterized by two small, early-diverging lineages successively branching off in the Oligocene, and the large split between section *Botryosambucus* and the remainder of species from section *Sambucus* occurred during the Late Oligocene (95 % HPD 15·8–33·8 Ma, mean age estimate 24·2 Ma). Divergence of both groups potentially occurred during the Miocene (Fig. 4).

For *Viburnum*, we observed an initial split during the Late Palaeocene, which resulted in two major lineages (a clade with species of section *Valvatotinus* and a clade with the remaining *Viburnum* species, respectively; Fig. 4). Whereas the clade containing species of *V.* section *Valvotinus* only started to diversify in the Oligocene (95 % HPD 21·6 – 43·5 Ma, mean age estimate 32·2 Ma), the latter clade had already diverged earlier during the Early to Middle Eocene (95 % HPD 41·2–58·6 Ma, mean age estimate 48·6 Ma).

### Ancestral area reconstruction

Hardly any ambiguous ancestral area reconstruction was observed, with relative probability values for the nodes of interest nearly always at least 20 % higher than the next ancestral area alternative. From the 126 nodes analysed, 111 had a relative probability value for a certain ancestral area above 50 %, whereas 79 nodes had a relative probability value above 90 %. According to our analysis, Adoxaceae as a whole, as well as the ancestral lineage towards *Sambucus*, *Adoxa*, *Sinadoxa* and *Tetradoxa*, have an East Asian (I) origin (Fig. 4). Therefore, stem lineages of both *Viburnum* and *Sambucus* have an East Asian (I) origin as well. At the time of initial divergence of *Sambucus*, a split can be observed into an East Asian (I) and an East Asian (I) or North American (C) lineage. For *Viburnum*, the earliest split results in an East Asian (I) and an East Asian (I) or combined East Asian–North American (CI) lineage (Fig. 4). The alternative ancestral area reconstruction analysis in which *V. clemensae* was constrained as sister species to extant *Viburnum* species provided results similar to those of the original analysis, thereby demonstrating that the different position of *V. clemensae* did not have a significant impact on the overall ancestral area analysis of *Viburnum* and *Sambucus*.

### Ancestral climate preference reconstruction

Plotting climate conditions onto the Adoxaceae phylogeny showed that the majority of lineages within *Sambucus* have a warm temperate preference, whereas the earliest diversification events within *Viburnum* show a cold temperate climate preference (Fig. 4). Nevertheless, several shifts from warm temperate to tropical or to cold temperate and *vice versa* have occurred within both genera. Our results reveal that the most recent common ancestor of *Sambucus* has a PP of 0·82 to be of warm temperate origin (0·13 cold temperate and 0·05 tropical origin). In contrast, the ancestor of *Viburnum* has a PP of 0·52 to be of cold temperate origin (0·36 warm temperate origin and 0·12 tropical origin). The alternative ancestral climate preference reconstruction analysis with *V. clemensae* constrained as sister to extant *Viburnum* species revealed similar but less pronounced results than in the original analysis: *Sambucus* has a PP of 0·80 to be of warm temperate origin (0·12 cold temperate and 0·08 tropical origin), whereas *Viburnum* has a PP of 0·45 to be of cold temperate origin (0·35 warm temperate and 0·21 tropical origin).

### Present climate preference reconstruction

The climatic niche of *Viburnum* largely overlaps with that of *Sambucus* based on present-day distribution data. *Viburnum* has a broader climatic niche, while *Sambucus* is more confined to regions with smaller extremes in precipitation and temperature throughout the year (Fig. S1). More specifically, *Sambucus* species occur in regions with less extreme temperatures during the warmest period of the year, with less extreme precipitation differences throughout the year and lower mean temperatures.

### Xylem specific conductivity (*K*_s_) measures

Mean (± s.e.) *K*_s_ of *S. nigra* was three times higher than in *V. lantana* (5·86 ± 1·3 versus 1·92 ± 0·26 m^2^ s^−^^1^ MPa^−^^1^ 10^−^^4^) for the stem segments representing ∼70 % open vessels. Standardizing sample length based on percentage of open vessels is important due to the shorter vessels in *Sambucus* compared with *Viburnum* (maximum vessel length 0·25 and 0·9 m, respectively). Mean (± s.e.) vessel diameter of both species was similar (25·86 ± 0·40 and 25·89 ± 0·27 μm, respectively), while the vessel density in *S. nigra* was significantly higher than in *V. lantana* (327 ± 2·2 and 215 ± 1·2 mm^−^^2^, respectively).

## Discussion

### Direction of the scalariform–simple perforation plate shifts within asterids is consistent with Baileyan trends, although reversals may occur

The wood anatomy of *Viburnum* perfectly fits with that of the Dipsacales outgroup Paracryphiales – characterized by an extremely large number of bars in scalariform perforation plates of up to 100 and more (Patel, 1973; Baas, 1975; Dickison and Baas, 1977) – and agrees with other early-diverging asterid lineages, thereby supporting its plesiomorphic nature (Fig. 2). Likewise, the evolution from scalariform towards simple perforation plates in Adoxaceae confirms the unidirectionality of the Baileyan trends, and represents one of numerous (at least ten) independent scalariform-to-simple transitions within asterids (Fig. 2; Baas and Wheeler, 1996; Lens *et al.*, 2007, 2008). However, the unidirectionality may not always apply in asterids: Fig. 2 suggests one reversal from simple to scalariform vessel perforations in the caprifoloid *Leycesteria*, and additional reversals may occur in species having mixed simple and scalariform perforations that can occur even within the same vessel or vessel element, as in Araliaceae (Oskolski and Jansen, 2009) or Ericaceae (Lens *et al.*, 2003, *2004b*, *c*). Nevertheless, the preponderance of the evidence suggests that scalariform-to-simple transitions significantly exceed reversals, and that the paucity of reversals makes them statistically indistinguishable from their complete absence in our present analysis (Fig. 3).

### The scalariform-to-simple perforation plate shift in Adoxaceae is driven by peak conductive rates

Our mean age estimates for the Adoxaceae are slightly older than those computed by Moore and Donoghue (2007) and Magallon *et al.* (2015), probably caused by differences in sampling, fossil calibration points and methods of analysis, but there is still an overlap between the 95 % confidence intervals of the two studies. We found that early diversification within *Viburnum*, showing scalariform vessel perforations with many (on average >20) bars, happened during the Late Palaeocene, which is about 15 million years earlier than the initial diversification of the simple-plated *Sambucus* clade during the Late Eocene (Fig. 4). Most likely, the difference in timing of early divergence between *Viburnum* and *Sambucus* allowed them to evolve in different niches, as supported by the ancestral climate preference reconstruction for Adoxaceae (Fig. 4): the earliest diversification events within *Viburnum* likely happened in cold temperate regions, while those of *Sambucus* occurred in warm temperate conditions. Thus, our analyses suggest that differences in temperature are associated with the scalariform-to-simple perforation plate transition in Adoxaceae.

The temperature scenario underlines our current ecological knowledge of wood anatomy, stating that species with scalariform perforations such as *Viburnum* typically occur in cool (and often mesic) regions that are characterized by low evaporative demands, while simple-plated species are often native to (seasonally) dry habitats (Carlquist, 1975; Baas *et al.*, 1983; Jansen *et al.*, 2004). This trade-off between type of vessel perforation and environment has been poorly investigated using xylem hydraulics. Based on the single-vessel technique, Christman and Sperry (2010) found experimental evidence that scalariform vessel perforations of diverse morphology double the lumen flow resistance, thereby impeding water flow much more than previously estimated based on models (e.g. Ellerby and Ennos, 1998; Schulte, 1999). Our hydraulic measures of *V.* *lantana* and *S.* *nigra* using standardized stem segments including ∼70 % open vessels and similar vessel diameters support this evidence. We found xylem-specific conductivity values three times higher in the simple-plated *S. nigra* compared with the scalariform-plated *V. lantana*, thereby confirming differences in *K*_s_ reported in the literature between *Sambucus* and *Viburnum* (25–33 and 13 m^2^ s^−^^1^ MPa^−^^1^ 10^−^^4^ respectively; Sperry *et al.*, 2005; Beikircher and Mayr, 2009). In other words, it is plausible to assume that selection for peak conductive rates has triggered scalariform-to-simple perforation plate transitions.

Although there is still controversy about the adaptive role of scalariform perforation plates, freezing temperatures may have been essential during the cold temperate climate in which the earliest diverging *Viburnum* species evolved (Fig. 4). Some authors claim that the closely spaced bars in the perforations can trap small freezing-induced air bubbles, thereby preventing detrimental levels of embolism when negative pressures in the transpiration stream increase (Zimmermann, 1983). However, other authors observed similar amounts of winter embolism in scalariform and simple-plated species of similar vessel size (Ewers, 1985; Davis *et al.*, 1999) and many scalariform-plated species occur in tropical montane regions that are frost-free (Jansen *et al.*, 2004).

Increasing vessel diameters may compensate for higher flow resistance in scalariform-plated species (cf. *Viburnum*, see below), but wider vessels make plants more vulnerable to freezing-induced embolism (Charrier *et al.*, 2013, 2014). As mentioned by Davis *et al.* (1999) and Zanne *et al.* (2014), 44 µm is the mean hydraulic diameter above which freezing-induced embolisms are becoming frequent at mild negative pressures, agreeing with the mean diameters observed of the temperate viburnums experiencing frost (32 µm) compared with the tropical lowland species (60 µm). In addition to vessel perforation plate morphology and vessel diameter, woody angiosperms have evolved an array of solutions to improve their water flow efficiency. Apart from the increased vessel connectivity in *Sambucus* compared with *Viburnum* (cf. Loepfe *et al.*, 2007; Sperry *et al.*, 2007; Lens *et al.*, 2011), other potential characters that could contribute to higher xylem-specific conductivity in *Sambucus* are thinner intervessel pit membranes (Choat *et al.*, 2008; Lens *et al.*, 2011, 2013) and higher ionic concentrations in xylem sap (Jansen *et al.*, 2011).

### *Viburnum*: out of the tropics?

The ancestral environment of *Viburnum* remains a matter of debate because of ambiguities in the relationships of the early diverging species. Based on our wider sampling within Adoxaceae, we found no evidence at all for a tropical ancestral habitat (PP 0·12) using our uniform methodology to code climate preferences for the species investigated, even in our analysis where we have constrained the tropical *V. clemensae* as root prior (PP 0·21). But in the latest *Viburnum* phylogeny with *V. clemensae* as root prior (Spriggs *et al.*, 2015), tropical lowland forests were favoured as the ancestral state, although with poor support.

Out of ∼165 *Viburnum* species, only ∼22 truly lowland tropical species exist today that grow up to 10–20 m tall (Kern, 1951), with several of them positioned in deep branches within the tree, suggesting that *Viburnum* has been slowly dying off in its potentially ancestral habitat (dying embers hypothesis; Spriggs *et al.*, 2015). According to this dying embers scenario, scalariform-plated species with decreased hydraulic efficiency growing in strongly competitive lowland forests would be outcompeted by more efficient, simple-plated eudicot lineages like *Inga* (Richardson *et al.*, 2001) and mahoganies (Koenen *et al.*, 2015) that have invaded these forests at a later stage (Sperry *et al.*, 2007). This would probably also explain the worldwide decrease of species with exclusively scalariform perforations from the Cretaceous (70–50 %) towards the current situation (18 %) (Wheeler and Lehman, 2009). This declining trend is even more pronounced in the modern tropical lowlands, where only a minor fraction of the tree species (<10 %) has exclusively scalariform perforations (Baas, 1976; Jansen *et al.*, 2004).

### Wood anatomical variation between *Viburnum* and *Sambucus* is pronounced, but their ecological niches greatly overlap

Although *Viburnum* and *Sambucus* are undeniably close relatives, their wood anatomy can hardly be more different from each other with respect to the variation observed across angiosperms. The key difference lies in vessel characters, but a whole range of additional anatomical patterns co-evolved with the scalariform-to-simple transition in perforation plates (cf. Frost, 1931; Kribs, 1935, 1937; Metcalfe and Chalk, 1950; see Table 1 for a list of anatomical characters that co-evolved with perforation plate morphology). Surprisingly, despite their marked wood anatomical divergence, both genera largely overlap in their climatic niche based on present-day distribution data (Fig. S1). This means that the basic wood anatomical bauplan in *Viburnum* and *Sambucus* had already been established during initial diversification, after which no major evolutionary shifts in wood characters have taken place.

Within *Viburnum*, we did find fine-scale differences between temperate and tropical species. The mature wood samples of the tropical *Viburnum* species studied, which are scattered into several major subclades (Fig. 4; Spriggs *et al.*, 2015), differ significantly at the 0·01 level in a number of quantitative features compared with the mature samples of the temperate relatives: the vessels are wider (on average 60 versus 32 µm) and less abundant (42 versus 110 mm^−^^2^), vessel elements and imperforate tracheary elements are longer (1310 versus 915 µm and 2120 versus 1340 µm, respectively) and rays are taller (1450 versus 670 µm; Table S1). This emphasizes that transitions to different climates within *Viburnum* have led to minor wood anatomical changes in specific cell types, as has been observed in other genera, such as *Symplocos* (van den Oever *et al.*, 1981), *Cornus* (Noshiro and Baas, 2000) or *Vaccinium* (Lens *et al.*, 2004*a*). Surprisingly, even the mean number of bars per scalariform perforation plate remains uniform based on our sampling (36 bars in tropical species versus 34 in temperate species). In other words, more efficient water transport in *Viburnum* seems to be caused by evolving wider vessels with more widely spaced bars in the perforation plates (following the Hagen–Poiseuille law) instead of developing fewer bars per plate. In addition to the more demanding hydraulic conditions in the tropics, the greater vessel diameters of tropical compared with temperate viburnums could also relate to the taller stature of the tropical species (Olson *et al.*, 2014).

### Concluding thoughts

In summary, asterids with simple vessel perforations have evolved many times independently from scalariform-plated relatives, although reversals may occasionally occur. Early diversification within *Viburnum*, characterized by ancestral scalariform perforation plates, occurred about 15 million years earlier than the initial diversification of *Sambucus* having simple perforations. The plesiomorphic perforation plates of *Viburnum* are in agreement with the frost-prone, cold temperate climates that were hypothesized in our ancestral climate preference reconstruction during the early diversification in the genus, while higher temperatures during early diversification of *Sambucus* may have triggered the evolution of simple vessel perforations, allowing improved long-distance water transport efficiency in the xylem. The much higher xylem-specific conductivity values of *S.* *nigra* compared with *V.* *lantana* confirm that the scalariform-to-simple perforation plate transitions are driven by selection acting on peak conductive rates. However, despite marked differences in wood anatomy and hydraulic conductivity between *Viburnum* and *Sambucus*, the ecological niches of the two genera based on present-day species distribution patterns largely overlap. Our study provides a first integrative approach to the underlying triggers behind the evolution of the scalariform-to-simple transition in perforation plates, but there is ample scope for further investigations of the convergent transitions within angiosperms. After Bailey’s pioneering work almost 100 years ago, we are starting to unravel the mechanism behind one of the most cited textbook examples in evolutionary plant anatomy.

## SUPPLEMENTARY DATA

Supplementary data are available online at www.aob.oxfordjournals.org and consist of the following. Note S1: species list of wood and DNA samples studied. Note S2: wood description of *Viburnum* and *Sambucus*. Note S3: phylogenetic relationships within *Viburnum* and *Sambucus*. Table S1: overview of selected wood anatomical characters within *Sambucus* and *Viburnum* (Adoxaceae). Table S2: sequence accession numbers and vessel perforation types for asterids. Figure S1: climate niche of *Sambucus* and *Viburnum.*

Supplementary Data
