# Supplementary material for: Scalariform-to-simple transition in vessel perforation plates triggered by differences in climate during the evolution of Adoxaceae
Source: Ann Bot. 2016 Aug 7;118(5):1043–56. doi: 10.1093/aob/mcw151 (PMC5055826; doi:10.1093/aob/mcw151)
Supplement: Supplementary Data [file supp_118_5_1043__index.html]

Scalariform-to-simple transition in vessel perforation plates triggered by differences in climate during the evolution of Adoxaceae — Supplementary Data 

# Scalariform-to-simple transition in vessel perforation plates triggered by differences in climate during the evolution of Adoxaceae

## Supplementary Data

files

- Supplementary Data - docx file
