# Supplementary material for: Scalariform-to-simple transition in vessel perforation plates triggered by differences in climate during the evolution of Adoxaceae
Source: Ann Bot. 2016 Aug 7;118(5):1043–56. doi: 10.1093/aob/mcw151 (PMC5055826; doi:10.1093/aob/mcw151)
Supplement: Supplementary Data [file supp_mcw151_aob-16239-s01.docx]

## *Annals of Botany* Supporting Information

Scalariform-to-simple transition in vessel perforation plates triggered by differences in climate during the evolution of Adoxaceae

Authors: Frederic Lens, Rutger A. Vos, Guillaume Charrier, Timo van der Niet, Vincent Merckx, Pieter Baas, Jesus Aguirre Gutierrez, Bart Jacobs, Larissa Chacon Dória, Erik Smets, Sylvain Delzon, and Steven B. Janssens

The following Supporting Information is available for this article:

Notes S1 Species list of wood and DNA samples studied.

Notes S2 Wood description of *Viburnum* and *Sambucus*.

Notes S3 Phylogenetic relationships within *Viburnum* and *Sambucus.*

Table S1 Overview of selected wood anatomical characters within *Sambucus* and *Viburnum* (Adoxaceae).

Table S2 Sequence accession numbers and vessel perforation types for asterids.

Fig. S1 Climate niche of *Sambucus* and *Viburnum.*

Notes S1. *Species list of wood and DNA samples studied.* Species list of wood samples studied (***in bold italics***) and DNA samples investigated *(in italics)* with reference to their locality, voucher data, and GenBank accession details of sampled taxa (atpB*-*rbc*L*, ITS, mat*K,* trn*K*, trn*S-G*). Missing sequences are indicated by a dash. Institutional wood collections are abbreviated according to Index Xylariorum (Stern, 1988). Wood specimens that were considered to be juvenile are marked with an asterisk. “Mature” means that the wood sample is derived from a trunk or mature branches, although the exact diameter of the wood sample could not be traced.

Adoxa moschatellina L., -, -, AF446990, U88194, EF490235, EF490235, EF490255; Sambucus adnata Wall. ex DC., Royal Botanic Garden Edinburgh, 20011059, HQ714333, HQ714441, HQ714358, HQ714386, HQ714414; Sambucus africana Standl., Botanic Garden Meise, Raynal 19216, HQ714334, HQ714442, HQ714359, HQ714387, HQ714415; Sambucus australasica (Lindl.) Fritsch, Australian National Botanic Garden, 9404563, HQ714335, HQ714443, HQ714360, HQ714388, HQ714416; Sambucus australis Cham. & Schltdl., Naturalis Biodiversity Center, Lindeman s.n., HQ714336, HQ714444, HQ714361, HQ714389, HQ714417; ***Sambucus australis***, Brazil (Santa Catarina, Bom Retiro), Reitz and Klein 7882 (MADw 18105), 50 mm; Sambucus caerulea Raf., Botanic Garden Meise, 10001803, HQ714338, -, HQ714363, HQ714391, HQ714419; **Sambucus caerulea***, USA (Oregon), Dechamps R (Tw 46253), 12 mm; Sambucus callicarpa Greene, Arboretum Kalmthout, 20030410, HQ714339, HQ714446, HQ714364, HQ714392, HQ714420; **Sambucus callicarpa**, USA (Mendocino County, California), Fritz E s.n. (SJRw 48758), mature; Sambucus canadensis L., Botanic Garden Meise, 20041199-29, HQ714340, -, HQ714365, HQ714393, HQ714421; **Sambucus canadensis^1^**, USA (Florida, Cocoa), Rhoads AS s.n. (MADw 9349), mature; **Sambucus canadensis^2^**, unknown, Barghoorn 7880 (Uw 9968); **Sambucus canadensis^3^**, USA (Florida), Stearns JL 1503 (MADw 9850), mature; Sambucus chinensis Lindl., Arboretum Provincial Domain Bokrijk, 20060323, HQ714341, HQ714447, HQ714366, HQ714394, HQ714422; Sambucus ebulus L., Botanic Garden Meise, 10003660, HQ714342, HQ714448, HQ714367, HQ714395, HQ714423; **Sambucus ebulus**, Botanic Garden Meise, 10003660 (living collections), 18 mm; Sambucus gaudichaudiana DC., Royal Botanic Garden Melbourne, s.n., HQ714343, HQ714449, HQ714368, HQ714396, HQ714424; Sambucus glauca Nutt. ex. Torr. & A.Gray, Botanic Garden Meise, Diran 3050, HQ714344, HQ714450, HQ714369, HQ714397, HQ714425; **Sambucus glauca**, USA (California), Green AN (Tw 22490), mature; Sambucus javanica Blume, Botanic Garden Meise, Kirkham 48, HQ714345, HQ714451, HQ714370, HQ714398, HQ714426; **Sambucus javanica**, Philippines, Stern WL 2246 (USw 31881), 20 mm; Sambucus kamtschatica E.L.Wolf., Botanic Garden Meise, s.n., HQ714347, -, -, HQ714400, HQ714428; Sambucus maderensis Lowe, Botanic Garden Meise, Leonard 6964, HQ714346, -, HQ714371, HQ714399, HQ714427; Sambucus mandshurica Kitag., Sir Harold Hillier Gardens and Arboretum, 1996-0452, -, HQ714464, HQ714385, HQ714413, HQ714440; Sambucus mexicana C.Presl ex DC., Botanic Garden University of Ghent, 20012460, HQ714348, HQ714453, HQ714373, HQ714401, HQ714429; **Sambucus mexicana**, Mexico (Chiapas), Breedlove D 9586 (MADw 23845), 35 mm; Sambucus microbotrys Rydb., Botanic Garden Meise, Bouharmont 31725, HQ714349, HQ714454, HQ714374, HQ714402, HQ714430; Sambucus nigra L., Botanic Garden Meise, 19830322, HQ714350, HQ714455, HQ714375, HQ714403, HQ714431; **Sambucus nigra^1^**, The Netherlands (Overijssel), University Utrecht s.n. (Tw 35334), 90 mm; **Sambucus nigra^2^**, France (Jura), Schweingruber FH (Tw 40709), 55 mm; **Sambucus nigra^3^**, Switzerland (Graubunden), Schweingruber FH (Tw 40865), 90 mm; **Sambucus nigra^4^**, Portugal (Baixa Alentejo), Dechamps R s.n. (Tw 43524), 25 mm; **Sambucus nigra^5^**, Poland; Warsaw Academy of Agriculture s.n. (Tw 47264), mature; **Sambucus nigra^6^**, Germany (Oberursel), Collector unknown (MADw 16845), mature; **Sambucus nigra^7^**, Italy (Florence), Botanical Garden of Florence s.n. (MADw 26373), mature; **Sambucus nigra^8^**, Austria (Steyr), Mayer W s.n. (MADw 39481), mature; **Sambucus nigra^9^**, Sweden, Collector unknown (USw 17050), mature; **Sambucus nigra^10^**, USA (Oregon, Portland); Binford T 615 (USw 19450), mature; **Sambucus nigra^11^**, Panama (Chiriqu); Collector unknown (USw 25862), mature; Sambucus palmensis Link, Botanic Garden Meise, Evrard 11434, HQ714351, HQ714456, HQ714376, HQ714404, HQ714432; **Sambucus palmensis**, Spain (Canary Islands, La Palma, Los Tilos); Collector unknown (USw 25862), mature; Sambucus peruviana Kunth, Botanic Garden Meise, Dellofer s.n., -, HQ714457, HQ714377, HQ714405, HQ714433; Sambucus pubens Michx., Holden Arboretum, 96-100, HQ714352, HQ714458, HQ714378, HQ714406, HQ714434; **Sambucus pubens**, Canada (Quebec), Drouin R (Tw 48554), 27 mm; Sambucus racemosa L., Botanic Garden Meise, 19852559, HQ714353, HQ714459, HQ714379, HQ714407, HQ714435; **Sambucus racemosa**, Switzerland (Graubunden), Schweingruber FH (Tw 40863), 55 mm; Sambucus sachalinensis Pojark., Arboretum Hof ter Saksen, 3909, HQ714354, HQ714460, HQ714380, HQ714408, HQ714436; Sambucus sieboldiana (Miq.) Blume ex. Schwer., Arboretum Hof ter Saksen, 19820127, -, -, HQ714381, HQ714409, -; **Sambucus sieboldiana**, Japan (Kagoshima prefecture, Takakuma forest), Toya Y s.n. (TWTw 13526), mature; Sambucus tigranii Troitsky, Botanic Garden Meise, 19851141, HQ714355, HQ714461, HQ714382, HQ714410, HQ714437; Sambucus wightiana Wall. ex. Wight & Arn., Royal Botanic Garden Edinburgh, 19942564, HQ714356, HQ714462, HQ714383, HQ714411, HQ714438; Sambucus williamsii Hance, Botanic Garden Meise, 19871920, HQ714357, HQ714463, HQ714384, HQ714412, HQ714439; **Sambucus williamsii**, Korea (Ulsan city, mount Shimburu-san), Noshiro S *et al.* s.n. (TWTw 23880), 15 mm; Sinadoxa corydalifolia C.Y.Wu, Z.L.Wu & R.F.Huang, -, -, AF446989, AF248611, AF446899, AF446899, EF490263; Tetradoxa omeiensis (H.Hara) C.Y.Wu, -, -, AF446991, AJ419710, AF446901, AF446901, EF490264; Viburnum acerifolium L., -, -, KJ796182, AY236170, EU749457, AY265160, EF490265; **Viburnum acerifolium***, USA (Pennsylvania), Nee M (Tw 51295), 8 mm; Viburnum adenophorum W.W.Sm., -, -, -, HQ591948, HQ591558, HQ591781, -; Viburnum atrocyaneum C.B.Clarke, -, -, -, HQ591950, HQ591559, HQ591782, HQ591820; Viburnum australe C.C.Morton, -, -, -, JQ805157, JQ805235, JQ805558, -; Viburnum awabuki K.Koch, -, -, -, JQ805210, JF956806, HQ591783, -; Viburnum ayavacense Kunth, -, -, -, JQ805162, JQ805238, JQ805561, -; Viburnum betulifolium Batalin, -, -, -, JF978998, JF956771, JQ805607, -; Viburnum bitchiuense Makino, -, -, -, JX049448, JX049451, JX049491, JX049495; Viburnum blandum C.V.Morton, -, -, -, HQ591952, HQ591562, HQ591785, -; Viburnum brachyandrum Nakai, -, -, -, -, HQ591563, HQ591786, HQ591821; Viburnum buddleifolium C.H.Wright, -, -, -, JQ805152, JX049458, JQ805551, JX049499; Viburnum burjaeticum Regel & Herd., -, -, -, JQ805153, JQ805231, JQ805553, JX049500; Viburnum calvum Rehder, -, -, -, HQ591955, HQ591565, HQ591788, JX049508; Viburnum carlesii Hemsl., -, -, KJ796184, AY265115, HQ591566, AY265161, HQ591823; **Viburnum carlesii**, Japan (Futamase, Bicchu Cho, Kawa kami Gun), Noshiro S *et al.* s.n. (TWTw 18378), 17 mm; Viburnum cassinoides L., -, -, KJ796185, HQ591956, HQ591567, HQ591789, HQ591824; Viburnum caudatum Greenm., -, -, -, HQ591957, -, HQ591790, HQ591825; Viburnum ciliatum Greenm., -, -, -, JQ805163, JQ805240, JQ805563, -; Viburnum cinnamomifolium Rehder, -, -, -, JQ805225, JQ805290, JQ805620, HQ591826; Viburnum clemensae J.Kern, -, -, KJ796186, AY265117, HQ591569, AY265163, EF490267; Viburnum colebrookeanum Wall. ex DC., -, -, -, HQ591959, HQ591570, HQ591791, -; Viburnum cordifolium Wall. ex DC, -, -, -, -, -, -, -; Viburnum coriaceum Blume, -, -, -, HQ591960, HQ591572, HQ591792, -; **Viburnum coriaceum**, India (Darjeeling), Indian Forestry Department 1878 (Lw), mature; Viburnum costaricanum (Oerst.) Hemsl., -, -, -, JQ805164, JQ587216, JQ805564, KF019909; **Viburnum costaricanum**, Costa Rica, Collector unknown (Uw 14850), mature; Viburnum cylindricum Buch.-Ham. ex. D.Don, -, -, KJ796187, JQ805151, JF956777, AY265165, EF490269; Viburnum davidi Franch., -, -, -, KF019821, KF019765, KF019951, KF019930; Viburnum dentatum Franch., -, -, KJ796188, AY265121, HQ591574, AY265167, HQ591827; **Viburnum dentatum**, USA (New York), Brett D s.n. (Tw 25998), 36 mm; Viburnum dilatatum Thunb., -, -, KJ796189, JF980314, JF956782, AY265168, HQ591828; **Viburnum dilatatum**, Japan, Noshiro S *et al.* s.n. (TwTw 21345), mature; Viburnum dicolor Benth., -, -, -, JQ805166, JQ805241, HQ591793, HQ591829; Viburnum divaricatum Benth., -, -, -, JQ805171, JQ805246, JQ805570, -; Viburnum edule (Michx.) Raf., -, -, -, AY265123, HQ591577, AY265169, EF490271; Viburnum elatum Benth., -, -, -, AY265124, HQ591578, AY265170, EF490272; Viburnum ellipticum Hook., -, -, -, AY265125, HQ591579, AY265171, HQ591830; Viburnum erosum Thunb., -, -, -, JQ805215, JQ805284, JQ805609, EF490273; Viburnum erubescens Wall., -, -, KJ796190, AY265127, HQ591581, AY265173, HQ591831; Viburnum farreri Stearn, -, -, -, AY265128, HQ591582, AY265174, EF490274; Viburnum flavescens W.W.Sm., -, -, -, HQ591962, HQ591583, HQ591794, JX049505; Viburnum foetidum Wall., -, -, -, JQ805217, JQ805286, JQ805610, JX049506; Viburnum furcatum Blume ex. Maxim, -, -, -, JQ805208, JQ805281, JQ805602, EF490275; Viburnum furcatum Blume ex. Maxim, Japan (Sudama Cho, Kita-koma Gun), Noshiro S *et al.* s.n. (TWTw 18597), 64 mm; ***Viburnum glaberrimum*** Merr., Philippines, Jacobs M 7434 (Lw), mature; Viburnum hallii (Oerst.) Killip & A.C.Sm., -, -, -, JQ805176, JQ805251, JQ805575, -; Viburnum hartwegii Benth., -, -, -, AY265130, HQ591586, AY265176, HQ591832; Viburnum hebanthum Wight & Arn., -, -, -, -, HQ591587, HQ591795, HQ591833; **Viburnum hebanthum**, unknown, unknown, mature; Viburnum hupehense Rehder, -, -, -, HQ591964, HQ591588, HQ591796, HQ591834; Viburnum ichangense (Hemsl.) Rehder, -, -, -, HQ591965, HQ591589, HQ591797, HQ591835; Viburnum integrifolium Hayata, -, -, -, JQ805219, KF019761, KF019947, KF019926; Viburnum jamesonii (Oerst.) Killip & A.C.Sm., -, -, -, HQ591966, HQ591591, HQ591798, HQ591836; Viburnum japonicum (Thunb.) C.K. Spreng., -, -, -, JQ805220, HQ591592, AY265177, HQ591837; Viburnum jucundum C.V.Morton, -, -, -, JQ805177, HQ591593, AY265178, HQ591838; Viburnum kansuense Batalin, -, -, -, JQ805156, JQ805234, JQ805557, EF490276; Viburnum koreanum Nakai, -, -, -, EF462983, -, EF490246, EF490277; Viburnum lancifolium P.S.Hsu, -, -, -, -, -, JQ805612, -; Viburnum lantana L., -, -, -, JX049450, JX049455, JQ805555, JX049503; Viburnum lantanoides Michx., -, -, KJ796192, AY265135, HQ591596, AY265181, EF490279; Viburnum lautum C.V.Morton, -, -, -, HQ591967, HQ591597, HQ591799, HQ591839; Viburnum lentago L., -, -, KJ796193, AY265136, HQ593493, AY265182, EF490280; Viburnum lobophyllum Graebn., -, -, -, JQ805221, HQ591600, AY265183, HQ591840; Viburnum loeseneri Graebn., -, -, -, HQ591968, HQ591601, HQ591801, -; Viburnum lutescens Blume, -, -, KJ796194, HQ591969, HQ591602, HQ591802, HQ591841; Viburnum luzonicum Rolfe, -, -, -, HQ591970, HQ591603, HQ591803, JX049507; Viburnum macrocephalum Fortune, -, -, -, EF462984, HQ591604, EF490247, HQ591842; Viburnum melanocarpum P.S.Hsu, -, -, -, AY265138, HQ591605, AY265184, HQ591843; Viburnum molle Michx., -, -, KJ796195, AY265139, HQ591606, AY265185, EF490281; Viburnum mongolicum (Pall.) Rehder, -, -, -, EF462985, HQ591607, EF490248, HQ591844; Viburnum nervosum D.Don, -, -, -, AY265118, HQ591571, AY265164, EF490268; Viburnum nudum L., -, -, -, AY265140, HQ591608, AY265186, EF490282; Viburnum obtusatum D.N.Gibson, -, -, -, JQ805183, JQ805256, JQ805579, -; Viburnum odoratissimum Ker. Gawl., -, -, -, JQ805212, HQ591609, JQ805606, HQ591845; **Viburnum odoratissimum**, Philippines, Jacobs M 7434 (Lw), mature; Viburnum oliganthum Batalin, -, -, -, HQ591971, HQ591610, HQ591804, HQ591846; Viburnum opulus L., -, -, KJ796196, HQ591972, JN896159, HQ591805, HQ591847; **Viburnum opulus**, The Netherlands (Delden forest), Collector unknown (Lw), 25 mm; Viburnum orientale Pall., -, -, -, EF462986, HQ591612, EF490249, EF490284; Viburnum parvifolium W.W.Sm., -, -, -, JQ805222, KF019763, KF019949, KF019928; Viburnum pichinchense Benth., -, -, -, JQ805184, JQ805260, JQ805584, -; Viburnum plicatum Thunb., -, -, KJ796197, AY265143, HQ591613, -, EF490285; **Viburnum plicatum**, Japan (Arimine, Ooyama Machi, Kami-niikama Gun), Noshiro S *et al.* s.n. (TWTw 21321), 45 mm; Viburnum propinquum Hemsl., -, -, -, EF462987, JQ805291, JQ805616, -; Viburnum prunifolium L., -, -, -, AY265144, HQ591615, AY265190, EF490286; **Viburnum prunifolium**, USA (Maryland), Wilson AF s.n. (USw 19493), mature; Viburnum punctatum Buch.-Ham. ex. D.Don, -, -, KJ796198, HQ591973, KF019748, KF019934, KF019913; **Viburnum punctatum** Buch.-Ham. ex. D.Don, Indonesia (Sumatra), Meijer 6314 (Lw), mature; Viburnum rafinesquianum Schult., -, -, -, AY265145, HQ591617, AY265191, HQ591849; Viburnum rhytidophyllum Hemsl., -, -, -, AY265146, JQ805233, JQ805556, HQ591850; Viburnum rigidum Vent., -, -, -, HQ591974, HQ591619, HQ591807, -; Viburnum rufidulum Raf., -, -, -, AY265147, HQ591620, AY265193, EF490287; **Viburnum sambucinum** Reinw. ex Blume, Indonesia (Sumatra), De Vogel 1333, mature; Viburnum scabrellum (Torr. & A.Gray) Chapm., -, -, -, JQ805190, JQ805262, JQ805586, -; Viburnum schensianum Maxim., -, -, -, HQ591975, HQ591622, HQ591808, HQ591851; Viburnum seemanii Graebn., -, -, -, JQ805193, JQ805263, JQ805588; Viburnum setigerum Hance, -, -, -, JQ805223, HQ591624, JQ805614, HQ591852; Viburnum sieboldii Miq., -, -, KJ796199, AY265149, HQ591625, AY265195, HQ591853; Viburnum stenocalyx (Oerst.) Hemsl., -, -, -, JQ805194, JQ805264, HQ591810, KF019912; Viburnum subalpinum Hand.-Mazz., -, -, -, HQ591979, HQ591627, -, -; Viburnum sulcatum (Oerst.) Hemsl., -, -, -, HQ591980, HQ591628, HQ591812, -; Viburnum suspensum Lindl., -, -, -, AY265151, HQ591629, AY265197, HQ591854; Viburnum sympodiale Graebn., -, -, -, JQ805209, JQ805282, JQ805603, EF490289; Viburnum taiwanianum Hayata, -, -, KJ796200, EF462989, JQ805292, JQ805618, HQ591855; Viburnum ternatum Rehder, -, -, -, HQ591981, HQ591632, HQ591813, HQ591856; Viburnum tinus L., -, -, KJ796201, JQ805228, HQ619809, JQ805617, HQ591857; **Viburnum tinoides var. venezuelense^1^** (Killip & A.C.Smith) Steyerm., origin unknown, collector unknown (Uw 35319), mature; **Viburnum tinoides var. venezuelense^2^**; Venezuela (Trujillo), Breteler FJ 4080 (WAGw), mature; Viburnum toronis Killip & A.C.Sm., -, -, -, JQ805199, JQ805271, JQ805594, HQ591858; Viburnum treleasei Gand., -, -, -, KF443803, HM850978, EF445122, -; **Viburnum tinus** L., origin unknown, Schweingruber F s.n. (L0086419), 23 mm, Viburnum triphyllum Benth., -, -, KJ796202, JQ805204, JQ805276, JQ805599, HQ591859; Viburnum urceolatum Siebold & Zucc., -, -, -, JQ805229, JQ805293, JQ805619, HQ591860; Viburnum utile Hemsl., -, -, -, AY265156, JF956809, AY265202, EF490291; Viburnum veitchii C.H.Wright, -, -, -, HQ591985, HQ591639, HQ591817, HQ591861; Viburnum villosum Sw., -, -, -, JQ805207, JQ805280, JQ805600, -; Viburnum wrightii Miq., -, -, -, JQ805224, JQ805289, HQ591818, HQ591862.

Notes S2. *Wood description of Viburnum and Sambucus.* Numbers without parentheses are ranges of means, while numbers between parentheses represent minimum or maximum values.

*Viburnum (Fig. 1a, 1c, 1e)*

Growth ring boundaries distinct in most temperate species (indistinct in *V. dilatatum*, *V. carlesii* and *V. tinus*), and indistinct to absent in the tropical species. Wood diffuse-porous. Vessels (4)-8-190-(215) mm^-2^; vessel grouping almost exclusively solitary; perforation plates exclusively scalariform, often with many bars (14)-19-48-(65). Intervessel pits opposite (4-8 µm in horizontal diameter) or scalariform (10-30 µm in horizontal diameter) or a mixture of both, nonvestured. Vessel-ray pits similar to intervessel pits in shape and size. Wall sculpturing present throughout vessel elements and tracheids of *V. acerifolium*, *V. carlesii*, *V. dilatatum*, and *V tinus*, sometimes only in tracheids of *V. dentatum*, and sometimes only confined to the tails of the vessel elements in *V. furcatum*, *V. hebanthum*, *V. sambucinum*, and *V. tinoides*. Tyloses not observed. Tangential diameter of vessels (10)-18-95-(120) µm; vessel elements (450)-665-1650-(2300) µm long. Ground tissue consists of true tracheids; pit borders 5-9 µm in horizontal diameter, concentrated in tangential and radial walls; tracheid length (650)-925-2600-(3100) µm; tracheids thin- to thick-walled to occasionally thick-walled. Axial parenchyma diffuse or diffuse-in-aggregates, with sometimes a light tendency to form short uniseriate bands in most tropical species; in (3)-5-8-(9) celled strands. Rays 1-2-4-(7)-seriate. Uniseriate rays abundant (often 3-10 uniseriate rays mm^-1^); height (100)-160-870-(1900) µm; uniseriate rays consisting of upright cells. Multiseriate rays generally 2-4-seriate; up to 5- or 6-seriate in *V. dentatum* and *V. plicatum*, up to 7-seriate in *V. costaricanum*; multiseriate ray height (150)-310-2940-(5300) µm high; multiseriate ray density (2)-3-6-(13) rays/mm; consisting of procumbent body ray cells and 1-10-(15) rows of upright marginal ray cells; multiseriate rays occasionally fused in most species; sheath cells indistinct in *V. dentatum*. Prismatic crystals and silica bodies not observed.

*Sambucus (Fig. 1b, 1d, 1f)*

Growth ring boundaries typically distinct, indistinct in *S. intermedia* and *S. javanica*, no growth rings observed in *S. ebulus*. Wood generally diffuse-porous, a tendency to semi-ring porosity in *S. callicarpa*, *S. nigra* (Tw 35334, Tw 40865) and *S. racemosa*, semi-ring porous in *S. caerulea*. Vessels (13)-22-190-(380) mm^-2^; vessel grouping usually distributed as a combination of radial multiples and clusters, sometimes ranging into tangential or oblique vessel patterns in *S. australis*, *S. callicarpa*, *S. glauca* and *S. nigra* (Tw 40709, Tw 40865, Tw 43524), vessels predominantly solitary in *S. javanica*, radial multiples most common in *S. intermedia*, clusters abundant in *S. glauca*; vessel outline angular to rounded; perforation plates simple, although a small proportion (< 5%) of scalariform perforations (usually < 5 bars) found in juvenile wood or in latewood of *S. caerulea*, *S. ebulus*, *S. maderensis*, *S. nigra* (most specimens studied), and *S. williamsii*. Intervessel pits alternate, pits 6-10 µm in horizontal diameter, nonvestured. Vessel-ray pits with much reduced borders, sometimes unilaterally compound. Wall sculpturing not observed. Tyloses occasionally present in *S. australis*, *S. caerulea*, *S. canadensis*, *S. glauca*, *S. javanica*, *S. maderensis*, *S. nigra* (most specimens), *S. palmensis*, *S. pubens*, *S. racemosa* and *S. williamsii*. Tangential diameter of vessels (20)-35-76-(120) µm; vessel elements (150)-300-685-(1350) µm long. Vascular tracheids typically present in the latewood of most species. Fibres with simple to reduced pit borders, 2-3 µm in horizontal diameter, concentrated in radial walls; fibre length (400)-525-1350-(1700) µm; fibres mainly thin-walled or thin- to thick-walled; fibres septate in *S. australis*, *S. canadensis*, *S. javanica*, *S. maderensis*, *S. nigra* (Tw 40709, USw 17050, USw 25862) and *S. palmensis*. Axial parenchyma scanty paratracheal, often scarce; in (2)-3-4-(5) celled strands. Rays 1-2-4-(6)-seriate. Uniseriate rays relatively scarce (often 1-2 uniseriate rays/mm); height (50)-120-440-(800) µm; uniseriate rays generally consisting of (square to) upright cells. Multiseriate rays generally 2-4-seriate; up to 5- or 6-seriate in *S. australis*, *S. canadensis*, *S. glauca*, *S. javanica*, *S. nigra* and *S. palmensis*; multiseriate ray height (150)-265-1485-(2600) µm high, but rays much taller in *S. ebulus* (> 8000 µm); multiseriate ray density (2)-4-7-(9) rays mm^-1^; consisting of procumbent body ray cells and mostly 1-2 rows of upright marginal ray cells, only upright ray cells in *S. ebulus* and *S. javanica*; multiseriate rays occasionally fused in *S. caerulea*, *S. canadensis*, *S. nigra*, and *S. sieboldiana*; sheath cells indistinct in most species. Prismatic crystals and silica bodies not observed.

Notes S3. *Phylogenetic relationships within Viburnum and Sambucus.* Our *Viburnum* topology supports previous work (Moore & Donoghue, 2007, Schmerler *et al.,* 2012; Clement *et al.,* 2014; Spriggs *et al.*, 2015), except for the position of *V. clemensae* and the position of section *Pseudotinus*. Studies by Donoghue and collaborators fix *V. clemensae* as root prior, making *V. clemensae* sister to the remaining *Viburnum* species. Our results, however, suggest that *V. clemensae* is sister to all *Viburnum* species excluding the earliest diverging *V.* section *Valvatotinus* *sensu* Winkworth & Donoghue (2005; BPP: 0.58). Donoghue and collaborators place section *Valvatotinus* as sister to *Pseudotinus*, whereas our analysis points to a closer relationship between section *Pseudotinus* and representatives of sections *Solenotinus* and *Tomentosa,* and *V. urceolatum and V. taiwanensis* (BPP: 0.97; Fig. 4). The position of section *Pseudotinus* is also doubtful in the latest phylogenies of Clement *et al.* (2014) and Spriggs *et al*. (2015), although most other clades are strongly supported. Our extended Adoxaceae sampling and the inclusion of *V. clemensae* as part of the ingroup sampling probably explains the small differences in topology.

In *Sambucus*, species of the section *Ebulus* *sensu* Hara (1983) are sister to the remainder of the genus (Fig. 4). The maximum parsimony tree of Eriksson & Donoghue (1997) based on ITS and morphology placed *S. caerulea* (section *Sambucus*) as sister to the rest of the genus, but this can refuted by our expanded dataset. A well-supported rest clade (BPP: 1.00) contains the monophyletic *Sambucus* section *Botryosambucus* (BPP: 1.00) and the paraphyletic section *Sambucus* *sensu* Bolli (1994), which was also recognised as paraphyletic in the study from Eriksson & Donoghue (1997). The sister species *S. australasiaca* and *S. gaudichaudiana* (section *Sambucus*) are sister to the remainder of this rest clade (BPP: 0.86), whereas *S. australis* (section *Sambucus*) is sister (BPP: 0.91) to the other species of section *Sambucus* (BPP: 0.99) and *Sambucus* section *Botryosambucus* (BPP: 1.00). The sister group relationship between the remaining species of section *Sambucus* and section *Botryosambucus* is hardly supported, however (BPP: 0.51; Fig 4).

Table S1. *Overview of selected wood anatomical characters within Sambucus and Viburnum (Adoxaceae).* Species in **bold** are tropical. Numbers between parentheses represent scarce characters or exceptional values. For specimens of the same taxon, superscript numbers after the species name refer to the order of the specimens as followed in the species list (see Appendix 1). Wood specimens that were considered to be juvenile are marked with an asterisk. DIFFUSE-POR = wood diffuse-porous, SEMI-RING-POR = wood semi-ring-porous, SOL = vessels mainly distributed as solitary entities, RAD MUL = vessel grouping often in radial multiples, CLUS = vessels often grouped in clusters, TANG OR OBL = vessels arranged in tangential or oblique patterns, VGI = vessel grouping index (measured by counting the number of vessels in 50 groups (solitary vessels are also considered as one group) and dividing the total number by 50, Carlquist, 2001), BARS SCAL PERF = number of bars in scalariform perforation plates, DIAM = tangential vessel diameter (µm), DENS = vessel density (mm^-2^), VEL = vessel element length (µm), FL/TL = fibre length (in case of *Sambucus*)/tracheid length (in case of *Viburnum*) (µm), SEPT F = septate fibres, MRW = multiseriate ray width (number of cells), MRH = multiseriate ray height (µm), MRD = multiseriate ray density (mm^-2^), URD = uniseriate ray density (mm^-1^).

| SPECIES | DIFFUSE-POR | SEMI-RING-POR | SOL | RAD MUL | CLUSTERS | TANG OR OBLI | VGI | BARS SCAL PERF | DIAM | DENS | VEL | VASC TRACH | FL/TL | SEPT F | MRW | MRH | MRD | URD |
| --- | --- | --- | --- | --- | --- | --- | --- | --- | --- | --- | --- | --- | --- | --- | --- | --- | --- | --- |
| *Sambucus australis* | + | - | - | (2-6) | 3-10-(20) | ± | 4.5 | / | (20)-56-(100) | (90)-114-(130) | (350)-495-(670) | - | (850)-1050-(1300) | + | 3-5 | (350)-610-(1100) | 4-6 | 1-2 |
| *S. caerulea** | - | + | - | 2-4-(8) | 3-10-(20) | - | 2.6 | (1-3) | (20)-43-(90) | (125)-165-220 | (180)-315-(410) | + | (350)-555-(730) | - | 2-4 | (150)-450-(1000) | 4-7 | 1-3 |
| *S. callicarpa* | - | ± | ± | 2-3-(5) | 3-15 | ± | 3.5 | / | (20)-50-(90) | (60)-105-(150) | (310)-455-(580) | + | (780)-965-(1270) | - | 2-4 | (250)-555-(1500) | 3-5 | 0-2 |
| *S. canadensis* | + | - | - | 2-4-(6) | (3-8) | - | 3.1 | (1-4) | (20)-54-(90) | (62)-82-(130) | (310)-430-(550) | - | (780)-990-(1150) | + | 3-5 | (250)-555-(1150) | 4-7 | 0-2 |
| *S. ebulus* | + | - | ± | 2-4-(6) | - | - | 2.3 | (3-10) | (25)-56-(80) | (88)-98-(106) | (410)-685-(1350) | - | (720)-915-(1200) | - | 2-3 | >8200 | 6-9 | 1-2 |
| *S. glauca* | + | - | - | - | 3-25 | + | 10,8 | / | (50)-76-(100) | (100)-120-(145) | (280)-390-(500) | + | (800)-1140-(1500) | ± | 3-6 | (300)-530-(950) | 4-6 | 0-3 |
| *S. intermedia* | + | - | ± | 2-5 | (3-10) | - | 3.5 | / | (30)-52-(80) | (100)-131-(185) | (240)-340-(420) | - | (400)-525-(760) | - | 2-4 | (300)-720-(1300) | 5-8 | 1-3 |
| ***S. javanica*** | + | - | + | (2-3) | - | - | 1.6 | / | (50)-75-(120) | (13)-22-(32) | (400)-540-(670) | - | (950)-1130-(1350) | + | 3-5 | (400)-1485-(2600) | 4-6 | 0-2 |
| *S. maderensis* | + | - | + | (2-3) | 3-5 | - | 2.0 | (1-6) | (35)-50-(65) | (58)-67-(80) | (500)-615-(800) | - | (950)-1170-(1300) | + | 2-4 | (200)-500-(850) | 4-5 | 0-1 |
| *S. mexicana* | + | - | - | 2-4-(8) | 3-10-(20) | - | 4.0 | / | (30)-55-(100) | (60)-110-(200) | (380)-570-(720) | - | (650)-940-(1300) | - | 2-4 | (250)-730-(1500) | 3-4 | 0-3 |
| *S. nigra*^1^ | - | ± | ± | 2-4 | (3-10) | - | 2.6 | / | (25)-56-(80) | (66)-80-(98) | (280)-390-(500) | + | (820)-1025-(1300) | - | (2)-3-4 | (150)-620-(1300) | 4-6 | 1-2 |
| *S. nigra*^2^ | + | - | ± | 2-5 | 3-10 | ± | 2.6 | (1-3) | (25)-44-(75) | (120)-163-(200) | (220)-345-(520) | + | (670)-850-(1000) | + | 2-4 | (200)-485-(900) | 5-9 | 1-3 |
| *S. nigra*^3^ | - | ± | - | 2-4 | 3-10 | ± | 4.1 | / | (20)-49-(80) | (65)-101-(150) | (270)-400-(470) | + | (650)-850-(1150) | - | 2-4 | (200)-500-(900) | 4-6 | 0-1 |
| *S. nigra*^4^ | + | - | - | (2-7) | 3-15 | ± | 5.0 | (1-3) | (20)-35-(50) | (140)-190-(380) | (220)-300-(380) | + | (550)-785-(970) | - | 3-5-(6) | (150)-600-(1500) | 5-7 | 2-5 |
| *S. nigra*^5^ | + | - | - | 2-4-(6) | 3-10 | - | 4.1 | (1-3) | (30)-59-(80) | (90)-114-(140) | (260)-490-(600) | + | (730)-1000-(1250) | - | 2-4 | (200)-510-(1000) | 5-7 | 0-2 |
| *S. nigra*^6^ | + | - | ± | 2-4-(5) | (3-10) | - | 2.9 | / | (20)-49-(80) | (86)-120-(160) | (280)-365-(450) | + | (650)-860-(1030) | - | 2-4 | (250)-590-(1500) | 5-6 | 1-2 |
| *S. nigra*^7^ | + | - | ± | 2-5-(7) | (3-10) | - | 2.3 | / | (25)-44-(70) | (100)-120-(140) | (340)-425-(510) | + | (730)-1015-(1280) | - | (2)-3-5 | (300)-530-(850) | 3-6 | 1-2 |
| *S. nigra*^8^ | + | - | - | 2-15 | 3-25 | - | 4.4 | (1-3) | (25)-56-(85) | (100)-120-(165) | (220)-320-(420) | + | (870)-1075-1340 | - | 3-5 | (300)-585-(1100) | 6-9 | 1-2 |
| *S. nigra*^9^ | + | - | ± | 2-5 | 3-10 | - | 2.5 | (1-5) | (25)-42-(60) | (65)-89-(120) | (200)-315-(450) | - | (650)-930-(1100) | ± | (2)-3-6 | (300)-510-(1100) | 5-7 | 1-2 |
| *S. nigra*^10^ | + | - | - | (2-5) | 3-10 | - | 2.7 | / | (25)-50-(70) | (60)-72-(100) | (200)-300-(400) | + | (650)-845-(1100) | - | 2-4 | (150)-440-(800) | 6-8 | 0-2 |
| *S. nigra*^11^ | + | - |  | (2-6) | 3-15 | + | 4.2 | (1-2) | (20)-44-(75) | (90)-111-(150) | (300)-370-(500) | - | (800)-1070-1300 | + | 2-4 | (200)-505-(1400) | 5-7 | 1-2 |
| *S. palmensis* | + | - | - | (2-4) | 3-25 | ± | 3.9 | / | (20)-49-(75) | (100)-135-(175) | (400)-615-(900) | ± | (900)-1215-(1450) | + | 3-5 | (350)-760-(1200) | 2-4 | 1-2 |
| *S. pubens* | + | - | ± | 3-10 | (2-4) | - | 2.7 | / | (20)-40-(70) | (110)-150-(180) | (300)-375-(450) | + | (700)-855-(1000) | - | 2-4 | (250)-420-(700) | 4-7 | 2-3 |
| *S. racemosa* | - | ± | - | 2-4-(6) | 3-15 | - | 4.7 | / | (20)-41-(60) | (105)-140-(180) | (150)-310-(450) | + | (500)-680-(760) | - | 2-4 | (200)-360-(700) | 4-7 | 1-3 |
| *S. sieboldiana* | + | - | ± | 2-4 | 3-15 | - | 3.2 | / | (30)-54-(90) | (82)-115-(160) | (220)-465-(550) | + | (850)-1350-(1700) | - | 2-4 | (250)-425-(900) | 5-7 | 1-2 |
| *S. simpsonii* | + | - | - | 2-4-(7) | 3-10 | - | 2.7 | / | (20)-52-(75) | (110)-133-(180) | (150)-360-(500) | ± | (650)-910-(1150) | - | 2-4 | (200)-445-(800) | 4-6 | 1-2 |
| *S. williamsii* | + | - | ± | 2-4 | - | - | 2.2 | (1-4) | (20)-35-(50) | (110)-132-(166) | (240)-325-(400) | + | (630)-820-(1080) | - | 2-3 | (150)-265-(450) | 4-7 | 1-2 |
| *Viburnum acerifolium** | + | - | + | - | - | - | 1.1 | (20)-39-(65) | (15)-23-(30) | (160)-190-(215) | (550)-950-(1250) | - | (850)-1275-(1500) | - | 2 | (400)-865-1250 | 2-4 | 10-14 |
| *V. carlesii* | + | - | + | - | - | - | 1.1 | (14)-20-(27) | (10)-18-(30) | (150)-168-(185) | (450)-665-(800) | - | (650)-925-(1100) | - | 2 | (200)-545-(1100) | 6-9 | 7-9 |
| *V. coriaceum* | + | - | + | - | - | - | 1.2 | (20)-35-(60) | (30)-40-(50) | (62)-70-(78) | (700)-1140-(1400) | - | (1500)-1830-(2300) | - | 2-3-(4) | (800)-1350-(1850) | 3-6 | 5-8 |
| *V. costaricanum* | + | - | + | - | - | - | 1.1 | (25)-40-(60) | (40)-61-(80) | (10)-13-(17) | (900)-1310-(1600) | - | (2200)-2590-(3000) | - | 4-7 | (1000)-2040-(3400) | 3-5 | 2-4 |
| *V. dentatum* | + | - | + | - | - | - | 1.3 | (25)-33-(45) | (20)-42-(55) | (70)-80-(85) | (500)-680-(900) | - | (1000)-1260-(1500) | - | 4-6 | (350)-840-(1700) | 2-6 | 4-6 |
| *V. dilatatum* | + | - | + | - | - | - | 1.2 | (28)-41-(55) | (25)-32-(40) | (60)-69-(82) | (600)-1100-(1600) | - | (1400)-1690-(2100) | - | 2-4 | (250)-770-(1250) | 3-5 | 4-9 |
| *V. furcatum* | + | - | + | - | - | - | 1.2 | (27)-43-(53) | (15)-33-(45) | (100)-115-(130) | (500)-825-(1150) | - | (1000)-1280-(1700) | - | 2-3 | (450)-600-(1150) | 7-13 | 3-4 |
| ***V. glaberrimum*** | + | - | + | - | - | - | 1.3 | (30)-38-(45) | (75)-95-(120) | (4)-8-(9) | (1050)-1480-(2300) | - | (1600)-2210-(2600) | - | 2-3-(4) | (500)-1140-(2510) | 2-3 | 3-4 |
| ***V. hebanthum*** | + | - | + | - | - | - | 1.3 | (15)-22-(28) | (40)-65-(80) | (24)-29-(38) | (950)-1220-(1700) | - | (1600)-2030-(2600) | - | 2-3 | (300)-510-(800) | 4-6 | 3-5 |
| ***V odoratissimum*** | + | - | + | - | - | - | 1.2 | (20)-37-(50) | (30)-40-(50) | (70)-80-(86) | (800)-1070-(1400) | - | (1300)-1800-(2200) | - | 2-3-(4) | (200)-1050-(1900) | 4-7 | 2-5 |
| *V. opulus* | + | - | + | - | - | - | 1.4 | (26)-37-(48) | (30)-37-(45) | (125)-146-(180) | (650)-1025-(1300) | - | (1200)-1390-1700 | - | 2 | (150)-490-(900) | 1-3 | 11-14 |
| *V. plicatum* | + | - | + | - | - | - | 1.2 | (23)-40-(55) | (25)-35-(40) | (54)-66-(80) | (650)-970-(1300) | - | (1200)-1535-(1950) | - | 3-4-(5) | (400)-755-(1600) | 3-6 | 10-14 |
| *V. prunifolium* | + | - | + | - | - | - | 1.1 | (14)-19-(26) | (25)-39-(50) | (46)-52-(62) | (700)-850-(1000) | - | (1000)-1275-(1500) | - | 2-(3) | (200)-310-(700) | 6-10 | 4-7 |
| *V. punctatum* | + | - | + | - | - | - | 1.1 | (13)-21-(28) | (35)-55-(70) | (34)-37-(42) | (700)-1030-(1500) | - | (1300)-1590-(1900) | - | 2-(3) | (450)-650-(1200) | 4-6 | 4-8 |
| *V. sambucinum* | + | - | + | - | - | - | 1.2 | (30)-47-(65) | (35)-53-(70) | (52)-60-(66) | (750)-1270-(1600) | - | (1600)-2000-(2400) | - | (2)-3-4-(5) | (800)-1760-(2600) | 3-5 | 3-4 |
| *V. tinoides* var. *venezuelense^1^* | + | - | + | - | - | - | 1.1 | (30)-38-(45) | (40)-61-(75) | (30)-40-(60) | (1000)-1600-(2100) | - | (1900)-2400-(2800) | - | 2-3 | (800)-1590-(2500) | 3-5 | 3-5 |
| *V. tinoides* var. *venezuelense^2^* | **+** | - | + | - | - | - | 1.2 | (35)-48-(65) | (50)-69-(90) | (37)-41-(44) | (1200)-1650-(2300) | - | (2000)-2600-(3100) | - | 2-3-(4) | (800)-2940-(5300) | 4-7 | 3-6 |
| *V. tinus* | + | - | + | - | - | - | 1.3 | (15)-24-(30) | (20)-27-(35) | (78)-89-(106) | (900)-1160-(1600) | - | (1050)-1410-(1600) | - | 2-(3) | (500)-870-(1600) | 3-7 | 6-7 |

Table S2. *Sequence accession numbers and vessel perforation types for asterids.* Sequence accession numbers for the markers used in the broad phylogenetic analysis of the asterids, and vessel perforation types used in the subsequent comparative analysis. Where applicable, the name of the collapsed clade in Fig. 2 is given.

| Species | Collapsed clade | rbcL | matK | rps16 | ndhF | Perforation plate type |
| --- | --- | --- | --- | --- | --- | --- |
| *Alseuosmia macrophylla* | Alseuosmiaceae | X87377 | AJ429378 |  | AJ238334 | scalariform |
| *Crispiloba disperma* | Alseuosmiaceae | X87382 | GQ983650 |  | AJ238338 | scalariform |
| *Wittsteinia vacciniacea* | Alseuosmiaceae | X87399 |  |  | AJ238345 | scalariform |
| *Helwingia japonica* | Aquifoliales | X94941 | AJ430195 | AJ431089 | AF130207 | scalariform |
| *Ilex cornuta* | Aquifoliales | AF471624 | GQ997309 | GQ997365 | GQ997315 | scalariform |
| *Phyllonoma ruscifolia* | Aquifoliales |  | AJ429377 | AJ431090 |  | scalariform |
| *Argophyllum nullumense* | Argophyllaceae | KM895989 | KM894822 |  |  | scalariform |
| *Corokia cotoneaster* | Argophyllaceae | L11221 | AY491646 |  | AF130182 | scalariform |
| *Pelliciera rhizophorae* | balsaminoid Ericales | AJ428893 | JQ589315 | AJ431014 | AF421069 | simple |
| *Pentamerista neotropica* | balsaminoid Ericales | AY725860 |  |  | AY725872 | simple |
| *Ruyschia phylladenia* | balsaminoid Ericales | AF303128 |  |  | AF303490 | simple |
| *Schwartzia costaricensis* | balsaminoid Ericales | JQ594913 |  |  | AF303493 | simple |
| *Souroubea exauriculata* | balsaminoid Ericales | AF303125 |  |  | AF303494 | simple |
| *Cordia dentata* | Boraginales | JQ590888 | JQ587086 | HQ286263 |  | simple |
| *Cordia nevillii* | Boraginales | HQ384923 |  | HQ385199 | HQ384860 | simple |
| *Cordia sagotii* | Boraginales | FJ037971 | FJ514773 |  |  | simple |
| *Columellia oblonga* | Bruniales | Y10675 |  | AJ431073 | AF060160 | scalariform |
| *Desfontainia spinosa* | Bruniales | Z29670 |  | AJ431074 | AJ011988 | scalariform |
| *Alangium kurzii* | Cornales | AF384108 | HQ427284 |  | JF321064 | scalariform |
| *Camptotheca acuminata* | Cornales | JQ280850 | JQ280882 | EU734472 | JF321071 | scalariform |
| *Cornus nuttallii* | Cornales | AF006833 | U96897 | FJ541998 | EU373590 | scalariform |
| *Curtisia dentata* | Cornales | L11222 | U96901 |  | JF321074 | scalariform |
| *Grubbia tomentosa* | Cornales | Z83141 | AF323184 |  | AJ400890 | scalariform |
| *Hydrangea arborescens* | Cornales | AF323186 | JF308692 | HE983410 | JF321089 | scalariform |
| *Nyssa sinensis* | Cornales | JF308651 | JQ280856 | EU734480 | JF321073 | scalariform |
| *Dipelta floribunda* | Dipelta | AJ420876 | FJ745399 |  |  | scalariform |
| *Dipelta yunnanensis* | Dipelta | AF446940 | AF446910 |  |  | scalariform |
| *Anginon rugosum* | early diverging Apiaceae | U50222 |  | AF110573 |  | simple |
| *Heteromorpha arborescens* | early diverging Apiaceae | JF265470 |  | DQ133866 |  | simple |
| *Steganotaenia araliacea* | early diverging Apiaceae | JQ025095 | EU214290 | AF110595 |  | simple |
| *Barnadesia caryophylla* | early diverging Asteraceae | L01887 |  |  | L39394 | simple |
| *Chuquiraga oppositifolia* | early diverging Asteraceae | EU841109 |  | EU547602 |  | simple |
| *Dasyphyllum argenteum* | early diverging Asteraceae | AY874428 |  |  | L39392 | simple |
| *Escallonia rubra* | Escalloniales | AJ419692 | AJ429365 | AJ431076 | AJ277383 | scalariform |
| *Polyosma cunninghamii* | Escalloniales | AF299091 | AJ429368 | AJ431079 | AJ419702 | scalariform |
| *Tribeles australis* | Escalloniales | AJ403010 | AJ429369 | AJ431080 | AJ429123 | scalariform |
| *Fouquieria columnaris* | Fouquieriaceae |  | EU628508 |  | AF207961 | simple |
| *Fouquieria splendens* | Fouquieriaceae |  | EU628509 |  | AJ236249 | simple |
| *Aucuba japonica* | Garryales |  | FN668800 | AJ431029 | AY725869 | scalariform |
| *Eucommia ulmoides* | Garryales |  | JF953701 | AJ431028 | AJ429113 | scalariform |
| *Garrya elliptica* | Garryales |  | AJ429319 | AJ431030 | AF147714 | scalariform |
| *Coffea arabica* | Gentianales | X81095 |  | AF004038 | AJ236290 | simple |
| *Gelsemium sempervirens* | Gentianales | HQ384909 | AJ429322 | AJ431033 | AF130170 | simple |
| *Posoqueria latifolia* | Gentianales | GQ981837 |  | FM204728 | AM949855 | simple |
| *Potalia resinifera* | Gentianales | AJ235816 |  |  | AJ235831 | simple |
| *Strychnos ludica* | Gentianales | DQ660668 | AB636278 | DQ660614 |  | simple |
| *Vangueria madagascariensis* | Gentianales | JF265645 |  | HM164229 | AJ130840 | simple |
| *Griselinia littoralis* | Griseliniaceae | AF307916 | AJ429372 |  |  | scalariform |
| *Griselinia lucida* | Griseliniaceae | L11225 | U58628 |  | AF130205 | scalariform |
| *Icacina mannii* | Icacina | AF206780 | HQ384577 | HQ385206 | AJ400888 | simple |
| *Icacina senegalensis* | Icacina | AJ428897 | AJ429313 | AJ431024 | AJ429111 | simple |
| *Abeliophyllum distichum* | Lamiales |  | EU281166 | AF225216 | DQ673262 | simple |
| *Avicennia germinans* | Lamiales |  | AF531771 | JN686517 | JN686621 | simple |
| *Buddleja asiatica* | Lamiales |  | AJ429346 | AJ431058 | AF027277 | simple |
| *Catalpa speciosa* | Lamiales |  | HE967373 | AJ609197 | DQ411407 | simple |
| *Halleria lucida* | Lamiales |  | HQ384528 | AJ609181 | AJ550569 | simple |
| *Oroxylum indicum* | Lamiales |  | HQ384520 | JN686524 | AF102635 | simple |
| *Osmanthus americanus* | Lamiales |  | EU409424 | AF225277 | DQ673285 | simple |
| *Schlegelia parviflora* | Lamiales |  | AJ429345 | AJ431057 | L36410 | simple |
| *Syringa reticulata* | Lamiales |  | JN590998 | AF225291 | JN591038 | simple |
| *Tabebuia ochracea* | Lamiales |  | JQ587051 | JN686527 | EF105038 | simple |
| *Tecoma stans* | Lamiales |  | HQ384522 | JN686528 | AF130145 | simple |
| *Thomandersia hensii* | Lamiales |  | FN773560 | FN794112 | AY919284 | simple |
| *Couratari calycina* | Lecythidaceae s.l. |  | JQ626524 |  | DQ388192 | simple |
| *Eschweilera parviflora* | Lecythidaceae s.l. |  | JQ626351 |  | DQ388223 | simple |
| *Lecythis chartacea* | Lecythidaceae s.l. |  | JQ626570 |  | DQ388209 | simple |
| *Napoleona vogelii* | Lecythidaceae s.l. | Z80173 | AJ429388 |  | DQ388164 | simple |
| *Oubanguia alata* | Lecythidaceae s.l. |  | KC627404 |  | DQ388165 | simple |
| *Lonicera canadensis* | Lonicera | HQ590165 | HQ593349 |  |  | simple |
| *Lonicera similis* | Lonicera | HM228507 | HM228463 |  |  | simple |
| *Lonicera webbiana* | Lonicera | JF942316 | JF954403 |  |  | simple |
| *Apodytes dimidiata* | Metteniusaceae | JF265292 | AJ429311 | AJ431022 | AJ429109 | scalariform |
| *Dendrobangia boliviana* | Metteniusaceae | FJ037991 | JQ626474 |  |  | scalariform |
| *Paracryphia alticola* | Paracryphiales |  | AJ429367 | AJ431078 | AJ429121 | scalariform |
| *Quintinia verdonii* | Paracryphiales |  | AJ429366 | AJ431077 | AJ238344 | scalariform |
| *Sphenostemon lobosporus* | Paracryphiales |  | GQ983660 | GQ983981 | GQ983668 | scalariform |
| *Pennantia corymbosa* | Pennantiaceae | AY188432 | AY188404 |  | AJ312949 | scalariform |
| *Pennantia cunninghamii* | Pennantiaceae | AJ494843 | AJ494845 |  | AJ312970 | scalariform |
| *Billardiera heterophylla* | Pittosporaceae |  | U58625 | DQ133886 | AJ236268 | simple |
| *Hymenosporum flavum* | Pittosporaceae |  | U58623 |  |  | simple |
| *Pittosporum tobira* | Pittosporaceae |  | HM850706 | AB178609 | AF130201 | simple |
| *Abrophyllum ornans* | Rousseaceae | X87375 | GQ983653 |  | AJ238333 | scalariform |
| *Carpodetus serratus* | Rousseaceae | Y08461 | AJ429383 |  | AJ238336 | scalariform |
| *Roussea simplex* | Rousseaceae | AF084477 | AJ429389 |  | AJ277384 | scalariform |
| *Sambucus adnata* | Sambucus | JF944173 | HQ714358 |  |  | simple |
| *Sambucus cerulea* | Sambucus | AJ420867 | HQ714363 |  |  | simple |
| *Sambucus chinensis* | Sambucus | JF944185 | HQ714366 |  |  | simple |
| *Manilkara zapota* | Sapotaceae | KF381146 | JN114734 | KM487487 | AY230696 | simple |
| *Pouteria guianensis* | Sapotaceae | FJ038183 | JQ413943 |  | DQ246711 | simple |
| *Kaliphora madagascariensis* | Solanales | HQ384922 | AJ429358 | HQ385198 | HQ384859 | simple |
| *Lycianthes heteroclita* | Solanales | AF397091 | JQ589231 |  | U72756 | simple |
| *Solanum arboreum* | Solanales | JQ594140 | JQ589580 |  | U47417 | simple |
| *Symphoricarpos albus* | Symphoricarpos | L11682 | GQ284948 |  | AF161291 | simple |
| *Symphoricarpos orbiculatus* | Symphoricarpos | AF446934 | GQ284970 |  | AF130195 | simple |
| *Viburnum clemensae* | Viburnum | HQ591714 | AY265163 | KJ795482 | HQ591648 | scalariform |
| *Viburnum opulus* | Viburnum | KP088910 | HQ591611 | KJ795492 | HQ591679 | scalariform |
| *Viburnum punctatum* | Viburnum | KF019790 | KF019934 | KJ795494 | KF019768 | scalariform |
| *Weigela florida* | Weigela |  | GU168657 |  | AF367421 | scalariform |
| *Weigela hortensis* | Weigela |  | AF446908 |  | AF447028 | scalariform |
| *Zabelia corymbosa* | Zabelia | HQ680727 | HQ693925 |  | HQ693948 | simple |
| *Zabelia triflora* | Zabelia | AF206727 | HQ693923 |  | HQ693946 | simple |
| *Zabelia tyaihyoni* | Zabelia | GQ983786 | HQ693922 |  | HQ693945 | simple |
| *Abelia chinensis* |  | HQ680737 | AY310461 |  | HQ693958 | scalariform |
| *Abelia floribunda* |  | HQ680733 | GU168641 |  | HQ693954 | scalariform |
| *Aralia stellata* |  |  |  | KF591529 | KF591502 | simple |
| *Aralidium pinnatifidum* |  | AF299087 |  | AJ431083 | AJ429126 | scalariform |
| *Cardiopteris quinqueloba* |  |  | AJ429310 | AJ431021 | AJ312963 | simple |
| *Cassinopsis ilicifolia* |  | AJ428896 | AJ429312 | AJ431023 | AJ429110 | scalariform |
| *Cheirodendron trigynum* |  | U50241 | U58606 | JX106148 | JX106029 | scalariform |
| *Clavija domingensis* |  | AF213818 |  |  | AY228595 | simple |
| *Clethra arborea* |  | AF421088 | HM850891 |  | AF421046 | scalariform |
| *Cyrilla racemiflora* |  | L01900 | AF380080 | AJ430995 | AF421051 | scalariform |
| *Diabelia serrata* |  | KP297679 | KP297520 |  | HQ693966 | scalariform |
| *Diervilla rivularis* |  |  | AJ429392 | AJ431105 |  | scalariform |
| *Diervilla sessilifolia* |  |  | AF446907 |  | AF060164 | scalariform |
| *Dimorphanthera megacalyx* |  |  | AF382757 |  | AF419725 | simple |
| *Diospyros lotus* |  | GU471699 | AB174993 |  | DQ924141 | simple |
| *Discophora guianensis* |  |  | JQ626375 |  | AJ312964 | scalariform |
| *Dracophyllum strictum* |  | GQ392925 | GQ392977 |  |  | scalariform |
| *Enkianthus campanulatus* |  | AB726259 | AB726241 |  | GU176746 | scalariform |
| *Erica carnea* |  |  | AY517908 | AJ430997 | AJ429105 | simple |
| *Hedera helix* |  | KM527348 | KJ204487 |  | AF130203 | simple |
| *Heptacodium miconioides* |  |  | GQ284983 | GQ984004 | AF283509 | simple |
| *Kalmia polifolia* |  | JN965615 | JN966342 |  | GU176733 | scalariform |
| *Kalopanax septolobus* |  | U50246 | KP089114 | GU055025 | GU054740 | simple |
| *Kolkwitzia amabilis* |  |  | AF446912 | GQ983989 | HQ693960 | scalariform |
| *Lasianthera africana* |  | KC628669 | KC627605 |  | AJ312948 | simple |
| *Leycesteria formosa* |  |  | HM850976 | GQ984000 | AF447022 | scalariform |
| *Lyonia ovalifolia* |  | AF124580 | U61305 |  |  | scalariform |
| *Mackinlaya confusa* |  | AY188426 | AF271741 |  |  | scalariform |
| *Mackinlaya macrosciadia* |  | U50247 | U58613 |  |  | simple |
| *Macropanax undulatus* |  | KJ440042 | KR531134 | GU055004 | GU054719 | scalariform |
| *Melanophylla alnifolia* |  |  | U58626 |  | AJ236244 | simple |
| *Myodocarpus fraxinifolius* |  |  | AF271747 | GQ984015 | GQ983678 | scalariform |
| *Needhamiella pumilio* |  | U80422 | AF539984 |  |  | simple |
| *Oncotheca balansae* |  | AJ131950 | AJ429320 | AJ431031 | AJ429114 | scalariform |
| *Oxydendron arboreum* |  | AF124583 | AF124562 |  |  | simple |
| *Pentaphragma ellipticum* |  | AJ419699 | AJ429387 | AJ431099 | AF130183 | scalariform |
| *Phelline lucida* |  | AJ238347 | AJ429388 | AJ431100 | AJ238343 | scalariform |
| *Pieris formosa* |  | AF124581 | U61303 |  |  | scalariform |
| *Plerandra insolita* |  |  |  | JX106161 | JX106042 | scalariform |
| *Prionotes cerinthoides* |  | U79743 | AF015642 |  |  | scalariform |
| *Saurauia tristyla* |  | KR529999 | EU310435 |  |  | scalariform |
| *Schefflera hypoleucoides* |  | AY753247 |  | KC952155 | KC952419 | scalariform |
| *Stewartia sinensis* |  | AF380061 | AF380106 |  |  | scalariform |
| *Styrax americana* |  | L12623 | KJ773191 |  | AF130215 | scalariform |
| *Symplocos caudata* |  | KJ688589 | KJ687836 |  |  | scalariform |
| *Torricellia tiliifolia* |  | AF299089 |  | AJ431087 | AJ429127 | simple |
| *Trevesia palmata* |  | KR530153 | KR531592 | KF591535 | KF591508 | simple |
| *Tupidanthus calyptratus* |  | U50259 | U58622 |  |  | scalariform |
| *Vaccinium membranaceum* |  |  | AF419711 |  | AF419755 | scalariform |
| *Vesalea coriacea* |  | KP297696 | KP297536 |  |  | scalariform |

Fig. S1: PCA plot showing climate niche of *Sambucus* and *Viburnum*. Current-day coordinates from a carefully selected *Viburnum* (30 sp.) and *Sambucus* (14 sp.) sampling showing the difference in climatic niches between both genera. *Viburnum* has a broader climatic niche, while *Sambucus* is more confined to regions with less extremes in precipitation and temperature throughout the year.

The *Viburnum* species used are *V. acerifolium, V. ayavakense, V. cassinoides, V. clemensae, V. costaricanum, V. dentatum, V. dilatatum, V. divaricatum, V. cylindricum, V. edule, V. ellipticum, V. erosum, V. erubescens, V. foetidium, V. furcatum, V. hartwegii, V. kansuense, V. lantanoides, V. lentago, V. lobophyllum, V. nervosum, V. nudum, V. obtusatum, V. odoratissimum, V. plicatum, V. rhytidophyllum, V. rufiludum, V. sympodiale, V. tinus,* and *V. utile.* The *Sambucus* species selected are *S. australis, S. caerulea, S. canadensis, S. ebulus, S. gaudichaudiana, S. javanica, S. mexicana, S. nigra, S. peruviana, S. pubens, S. racemosa, S. sieboldiana, S. williamsii* and *S. wrightiana.* Abbreviation BIOCLIM variables: TS: Temperature seasonality, TAR: Temperature annual range, MDR: Mean diurnal range, PWarQ: Precipitation of the warmest quarter, PWetQ: Precipitation of the wettest quarter, PS: Precipitation seasonality, MTWarQ: Mean temperature of warmest quarter, AMT: Annual mean temperature, MTColQ: Mean temperature of coldest quarter, MTColM: Mean temperature of coldest month, MTWarM: Mean temperature of warmest month, MTWetQ: Mean temperature of wettest quarter, MTDQ: Mean temperature of driest quarter, PWetM: Precipitation of wettest month, MTColM: Minimum temperature of coldest month, AP: Annual precipitation IS: Isothermality, PColQ: Precipitation of coldest quarter, DQ: Precipitation of driest quarter, PDM: Precipitation of driest month

**
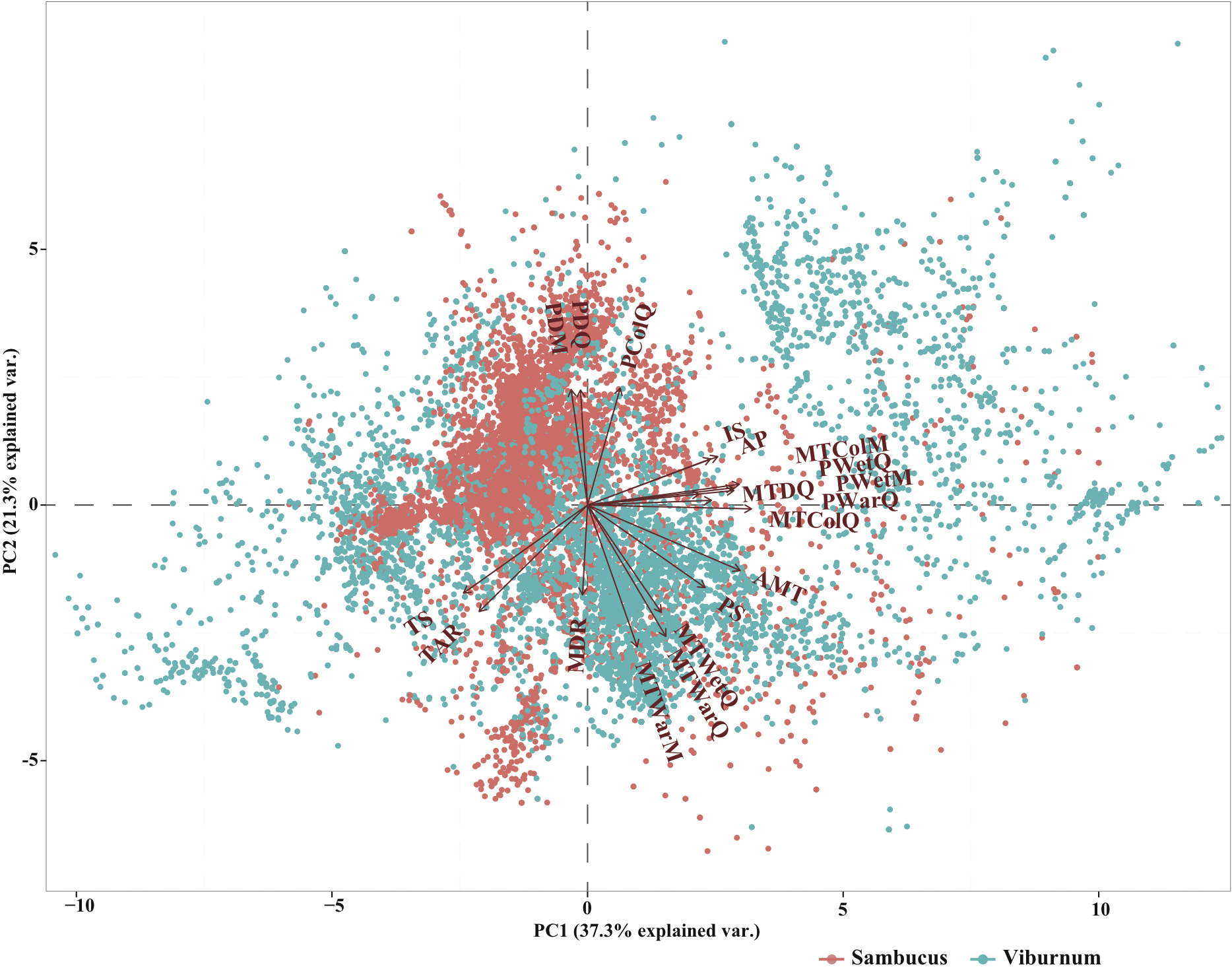
**

**References**

**Carlquist S. 2001.** *Comparative wood anatomy: Systematic, ecological, and evolutionary aspects of dicotyledon wood*, 2nd ed. Berlin, Germany: Springer-Verlag.

**Stern WL. 1988.** Index Xylariorum. Institutional wood collections of the world, 3rd ed. *International Association of Wood Anatomists Bulletin, new series,* **9**, 204-252.
